# Synergistic Insecticidal Activity of Plant Volatile Compounds: Impact on Neurotransmission and Detoxification Enzymes in *Sitophilus zeamais*

**DOI:** 10.3390/insects16060609

**Published:** 2025-06-09

**Authors:** Leidy J. Nagles Galeano, Juliet A. Prieto-Rodríguez, Oscar J. Patiño-Ladino

**Affiliations:** 1 Departamento de Química, Facultad de Ciencias, Universidad Nacional de Colombia, Sede Bogotá, Bogotá 111321, Colombia; lnagles@unal.edu.co; 2 Departamento de Química, Facultad de Ciencias, Pontificia Universidad Javeriana, Bogotá 110231, Colombia; juliet.prieto@javeriana.edu.co

**Keywords:** *Sitophilus zeamais*, fumigant toxicity, contact toxicity, synergistic interactions, nervous and detoxifying systems, acetylcholinesterase

## Abstract

*Sitophilus zeamais*, a major pest of stored grains, causes significant post-harvest losses and challenges effective control. While synthetic insecticides pose risks of resistance and toxicity, essential oils (EOs) offer a safer alternative. However, the insecticidal potential of their individual volatile constituents (VCs) remains largely unexplored. This study evaluated the insecticidal activity of 51 EO-derived volatile compounds (VCs) against *S. zeamais*, identifying the most toxic ones, optimizing 15 synergistic mixtures, and assessing their effects on key insect enzymes. A structure–activity relationship (SAR) analysis determined functional groups associated with insecticidal activity, while a cluster analysis pre-selected 29 ternary mixtures, later refined using response surface methodology (RSM). Additionally, enzymatic assays explored their impact on detoxification and nervous system enzymes, providing insights into potential mechanisms of action. Among the 51 VCs tested, 37 exhibited significant toxicity, with 11 acting as fumigants and 13 displaying contact toxicity. Monocyclic monoterpenoids with ketone or alcohol functional groups and exocyclic unsaturation demonstrated the highest insecticidal activity via both exposure routes. Notably, pulegone enantiomers were particularly effective (LC_50_ < 0.1 mg/L, LD_50_ < 7.5 µg/adult). Among the optimized mixtures, 10 displayed strong insecticidal effects, 8 were active through both routes, and 5 exhibited synergistic fumigant interactions. The most effective formulations were M2 (R-pulegone + S-pulegone + S-carvone, LC_50_ 0.48 mg/L) and M20 (isopulegone + δ-3-carene, LC_50_ 2.06 mg/L), showing the strongest fumigant and synergistic effects, respectively. Enzymatic assays revealed that while some compounds mildly inhibited GST and CAT, others, such as δ-3-carene (IC_50_ 0.19 mg/L), significantly inhibited AChE. Five mixtures exhibited synergistic neurotoxicity, with M20 (IC_50_ 0.61 mg/L) and M12 (IC_50_ 0.81 mg/L) emerging as the most potent AChE inhibitors. These findings highlight the potential of plant-derived volatile compounds as bioinsecticides, leveraging synergistic interactions to enhance efficacy, disrupt enzymatic pathways, and mitigate resistance.

## Simple Summary

This research addresses the critical issue of *Sitophilus zeamais* infestation in stored grains, a significant threat to food security. The conventional use of synthetic insecticides, while effective, presents significant drawbacks, including high costs, toxicity, and the development of resistance. Therefore, this study explores plant-derived phytosanitary agents, specifically essential oils (EOs), as safer and more sustainable alternatives. Previous studies have demonstrated the insecticidal properties of various EOs against *S. zeamais*; however, the understanding of their active constituents and synergistic interactions remains limited. To bridge this gap, this study systematically analyzed 51 plant-synthesized volatile compounds (VCs) derived from EOs, identifying 37 with potent insecticidal activity. Additionally, 15 optimized mixtures were designed, revealing synergistic combinations that enhance efficacy. Furthermore, this study evaluated the impact of these compounds on key insect enzymes, uncovering potential neurotoxic effects and dose-dependent reductions, suggesting a multitarget mode of action. Overall, this research provides valuable insights into refining plant-based insecticides to effectively combat grain pests and enhance food security.

## 1. Introduction

Cereals are a vital component of global nutrition and represent an essential source of carbohydrates, proteins, fibers, phytocompounds, minerals, and vitamins [1,2]. While many cereals can be stored for extended periods without significant loss of nutritional value, post-harvest storage is often compromised by substantial quality deterioration, primarily due to insect pest infestations [3,4]. *Sitophilus zeamais* Motsch (Coleoptera: Curculionidae) is a cosmopolitan primary pest that negatively impacts the nutritional quality and sensory properties of stored grains such as maize, wheat, rice, and sorghum [5,6]. Chemical insecticides are commonly used to control *S. zeamais*; however, many exhibit low selectivity and high toxicity. The indiscriminate use of these products has led to environmental contamination and the emergence of resistant insect populations [7,8]. Therefore, the development of effective and environmentally safe alternatives remains a priority in agricultural research.

Essential oils (EOs) have emerged as promising candidates for pest control due to their favorable physicochemical properties and documented insecticidal activity. These oils are typically complex mixtures of volatile secondary metabolites that exert insecticidal effects through diverse modes and mechanisms of action, thereby reducing the likelihood of resistance development [9,10,11,12,13,14,15,16,17]. Numerous studies have demonstrated the potential of EOs to control *S. zeamais*, which include different toxic (contact, ingestion, and fumigation) and behavioral (repellent, feeding deterrence, and inhibition of oviposition and growth) effects [17,18,19,20,21]. EOs can alter the metabolic, physiological, and behavioral functions of insects through various mechanisms, which have been classified as direct or metabolic [14,17]. Direct effects primarily target the nervous system, including the modulation of GABA and octopamine receptors and inhibition of acetylcholinesterase (AChE), whereas metabolic effects influence detoxifying enzymes, hormonal pathways, and developmental processes [12,13,22,23].

Despite the growing interest in the insecticidal properties of EOs, studies that specifically investigate the insecticidal activity and behavioral effects of their individual chemical constituents against *S. zeamais* remain limited [13,16,22,23,24,25,26,27,28]. Some studies have described the insecticidal properties of specific chemical constituents and have established preliminary structure–activity relationships, suggesting that the α,β-unsaturated carbonyl group in monocyclic monoterpenes plays a key role in insecticidal activity [27,28]. This study investigates the insecticidal and synergistic effects of 51 plant-derived volatile compounds against *S. zeamais* and evaluates their impact on the insect’s detoxification and nervous system enzymes.

## 2. Materials and Methods

### 2.1. Chemicals

The plant-derived volatile compounds (VCs) used in this study were pre-selected based on their presence in bioactive EOs with known activity against *S. zeamais*. Of the 51 compounds tested, 48 were commercially sourced, as detailed in Supplementary Materials Table S1. Estragole and piperitone were isolated via flash chromatography from the EOs of *Artemisia dracunculus* and *Piper aduncum*, respectively. Isopulegone and pulegone were synthesized from commercial *1R*,*2S*,*5R*-isopulegol (Sigma-Aldrich^®^, Saint Louis, MO, USA) using adapted methodologies from the literature [23]. The isolated and synthesized compounds were characterized by NMR and GC-MS (Tables S1–S4 and Figures S1–S6). In the in vitro assays, acetylcholinesterase (AChE) from electric eel (376 U/mg), glutathione S-transferase (GST) from equine liver (140 U/mg), catalase (CAT) from bovine liver (4918 U/mg), acetylthiocholine iodide (AThChI), 5,5′-dithiobis-(2-nitrobenzoic acid) (DTNB), hydrogen peroxide (H_2_O_2_), potassium dichromate (K_2_Cr_2_O_7_), 1-chloro-2,4-dinitrobenzene (CDNB), and reduced glutathione (GSH) were used, all obtained from Sigma-Aldrich^®^, Saint Louis, MO, USA.

### 2.2. Insecticide Activity

#### 2.2.1. Insects

The insects used in this study were *Sitophilus zeamais* Motschulsky (Coleoptera: Curculionidae), identified by the Instituto Colombiano Agropecuario (ICA) under report number R3823M0000425. The breeding colony was maintained on previously washed and dried porva corn in a growth chamber under controlled conditions: darkness, 70 ± 5% relative humidity, and 28 ± 2 °C. The adult insects used in all the experiments were 6 to 10 days post-emergence.

#### 2.2.2. Fumigant Toxicity Assay

Fumigant activity was evaluated using the vial-in-vial method with slight modifications [21]. Each compound was applied at a concentration of 150 mg/L of air (650–730 µM) to Whatman^®^ No. 1 filter paper disks, Boeco, Hamburg, Germany (2.0 cm diameter) using a micropipette. The treated disks were affixed to the inner surface of the screw cap of 22 mL glass vials. To prevent direct contact between the insects and the compound, a layer of 15% polytetrafluoroethylene (PTFE) was placed over the vial before introducing ten insects. The vials were then sealed tightly to create an enclosed exposure chamber and incubated at 28 ± 2 °C and 70 ± 5% RH. Insect mortality was assessed after 24 h, and corrected mortality percentages were calculated using Abbott’s formula [29] as follows:

(1) $$\%Mortality=\frac{\%M_{t}-\%M_{c}}{100-\%M_{c}}\times 100$$

where *M_t_* represents mortality in the treatment group and *M_c_* represents mortality in the negative control. Nuvan^®^ 50 E.C., which contains dichlorvos as the active ingredient, was used as a positive control at a concentration of 50 mg/L of air under the same experimental conditions. All treatments were performed in two independent experiments, each with five replicates. Compounds that induced mortality rates of 60% or higher were subsequently tested at varying concentrations using vials of different volumes (22, 140, 270, and 518 mL) to eliminate the use of solvents. This setup enabled the estimation of lethal concentrations (LC_50_ and LC_90_) through Probit analysis using SPSS Statistics 25 (IBM^©^, New York, NY, USA) [30].

#### 2.2.3. Topical Contact Toxicity Assay

A topical application method was used as described in the literature, with minor modifications [11,18]. Groups of 10 insects, previously placed in Petri dishes, were immobilized by exposure to low temperatures (0 °C). An initial dose of 50.0 µg per adult was applied topically to the dorsal thorax of each insect using aliquots of 0.20 µL from 0.25 mg/µL solutions in *n*-hexane. Applications were made using a Microliter #7001 Knurled Hub Syringe (PN: 80100, Hamilton). The solvent (*n*-hexane) served as the negative control, while Hawker 25 E.C., which contains cypermethrin as the active ingredient, was used as the positive control. Treated and control insects were transferred to 22 mL glass vials and maintained in a climate-controlled chamber at 28 ± 2 °C and 70 ± 5% RH. All treatments were performed in two independent experiments, each with four replicates. Insect mortality was assessed after 24 h, and mortality percentages were calculated using Abbott’s correction formula (Equation (1)). Compounds causing ≥60% mortality were further evaluated at varying concentrations to estimate their LD_50_ values using Probit analysis (SPSS Statistics, version 25.0) [30].

#### 2.2.4. Cluster Statistical Analysis

The response variable matrix included lethality parameters (LC_30_, LC_50_, LC_90_, and slope) and the number of exposure cycles [31]. Inter-group distances were calculated using Ward’s method, and the optimal number of clusters was set to K = 3 based on the Hubert and D indices [32]. Two clustering methods were applied: a k-means partitioning algorithm (an unsupervised, centroid-based approach) for grouping compounds by chemical structure, and a hierarchical AGNES algorithm (agglomerative nesting) based on variable similarity for finer cluster formation. The k-means method was used for fumigant toxicity analysis because of its suitability for handling qualitative variables, while the AGNES method yielded a more appropriate distribution of volatile compounds (VCs) in the contact toxicity analysis. All the statistical analyses were conducted using RStudio version 3.6.3 [31,33]. Additionally, selected physicochemical and structural attributes—such as carbon skeleton, functional group type and position, compound class, biosynthetic origin, vapor pressure, and partition coefficient—were manually assigned as categorical variables to support a preliminary structure–activity relationship (SAR) analysis (Table S2).

### 2.3. Design of Mixtures of Bioactive Volatile Metabolites

#### 2.3.1. Pre-Design of the Mixtures

The cluster analysis results were used to define the composition of ternary mixtures. Initially, combinations were formulated using the most active volatile compounds (VCs) within the same cluster. Additional mixtures were subsequently designed by combining VCs from different clusters. The selection criteria for these combinations included insecticidal efficacy and the structural features of the compounds, such as carbon skeleton, biosynthetic origin, and functional group type and position. This strategy aimed to enhance insecticidal effects on a single molecular target, generate multitarget mixtures, or reduce the required doses of bioactive VCs (Supplementary Materials, Tables S6 and S7). Moreover, some mixtures were designed based on the known chemical composition of bioactive essential oils (EOs), especially when two or more promising components were identified within the same EO, incorporating concepts from chemical ecology. Fumigant toxicity mixtures were labeled M1 to M20 (Table S6), and contact toxicity mixtures were designated MC1 to MC9 (Table S7).

#### 2.3.2. Response Surface Model (RSM)

Following the pre-design stage, the components and binary combinations of each mixture were tested in 1:1 ratio, using concentrations or doses below the LC_90_ or LD_90_ of the most active compound in each ternary mixture. A response surface model (RSM) based on a {3,2} simplex lattice was applied, considering the intended route of exposure (fumigant or contact) [34].

Experimental data for all mixtures fitted to a quadratic model (Equation (2)), which was significant in all cases. The normality of residuals was verified using the Anderson–Darling test and normal probability plots. Model adequacy was evaluated by ANOVA [35].

(2) $$y=b_{1}\times x_{1}+b_{2}\times x_{2}+b_{3}\times x_{3}+b_{12}\times x_{1}\times x_{2}+b_{13}\times x_{1}\times x_{3}+\;b_{23}\times x_{2}\times x_{3}$$

Contour plots and Cox effect traces were generated to visualize the response surface in relation to a reference mixture (centroid, as estimated by the model), while keeping the relative proportions of the remaining two components constant. This analysis aimed to identify mixtures with maximal insecticidal activity against ten adult insects, focusing on predictions involving at least two components [35,36,37].

#### 2.3.3. Effect of Insecticidal Interaction

Mixtures predicted by the RSM were prepared based on mole fractions. Each predicted combination was tested at the LC_50_ (fumigant) or LD_50_ (contact) of the most active component, using the mode of action associated with the pre-design (topical or fumigat toxicity method) [37,38]. Mixtures producing ≥50% mortality were further evaluated to determine their toxic effects (LC_50_ or LD_50_) [30]. Four replicates were performed for each treatment and control across two independent experimental runs.

Interaction types were assessed using the median-effect model based on the mass action law, implemented in CompuSyn software 1.0 (additive model) [39,40] The combination index (CI) was calculated (Equation (3)) using doses or concentrations corresponding to 30%, 50%, 75%, and 90% lethality levels (Probit estimation). Dose–effect curves were represented as linear plots (Figures S7 and S8) [40,41]. This model assumes that the combined compounds act through mutually exclusive mechanisms, since all components were designed for the same exposure route [38]. Interactions were classified as synergistic if CI < 1, additive if CI = 1, and antagonistic if CI > 1 [41].

(3) CI=(D)i(Dx)i+(D)j(Dx)j=(D)iCL50i(fax)i/(1−(fax)i)1mi+(D)jCL50j(fax)j/(1−(fax)j)1mj+…

where $D_{i}$ is the concentration or dose of compound i, $D_{j}$ is the concentration or dose of compound j, $f_{ax}$ is the fractional toxicity at x% mortality, m is the sigmoidity coefficient of the dose–response curve, and ${\left(D_{x}\right)}_{i}$ is for $D_{i}$ “alone”, which inhibits x% of the system [41].

Dose reduction indices (DRIs) were also calculated to estimate how much the dose of each compound in a synergistic combination could be reduced to achieve a 50% effect (Equation (4)). DRIs were interpreted as favorable when >1, neutral at 1, and unfavorable when <1. Values above the log DRI = 0 line indicate favorable dose reductions [40].

(4) (IRD)i=CL50i(fax)i/(1−(fax)i)1mi(D)i

### 2.4. Enzymatic Effects of the Most Active Mixtures and Their Components

Enzymatic assays were performed in the assay buffer, consisting of 0.1 M potassium phosphate buffer (pH 7.2). The compounds and mixtures evaluated in the in vitro assays were prepared in the same buffer supplemented with 0.5% (*v*/*v*) Triton X-100 (Sigma-Aldrich^®^, Saint Louis, MO, USA).

#### 2.4.1. Enzyme Extraction from *S. zeamais*

A modified version of the method described by Oviedo et al. was used [11,42]. Adults of *S. zeamais* (8 g; ~3125 insects) were frozen in liquid nitrogen, homogenized in a 1:10 ratio with assay buffer, and centrifuged at 13,000× *g* for 60 min at 4 °C. The supernatant was filtered, and 20 mM benzamidine was added. The extracts were stored in 5 mL aliquots at −20 °C. Preliminary assays were performed to quantify the specific activity of AChE (0.02–1.00 U/mL), GST (0.1–0.8 U/mL), and CAT (0.1–25 U/mL), and to optimize assay conditions (enzyme dilution: 1/8 to 1% *v*/*v*; substrate concentrations: 2 × km or within assay linearity). Each experiment was performed independently three times, with three technical replicates per experiment [42,43].

#### 2.4.2. Protein Quantification

Protein content was measured using the Bradford method [44,45], with bovine serum albumin (BSA; 5–60 µg/mL) as the standard. Enzymatic extract (50 µL, 12.5% *v*/*v*) and 200 µL of Bradford reagent were mixed in a 96-well plate, shaken for 1 min, and incubated for 9 min at room temperature in the dark. Absorbance was measured at 595 nm. Each extract was analyzed in triplicate across three independent assays.

#### 2.4.3. GST Activity Assay

A modified version of Habig’s method was used [46,47]. The protein extract (50 µL; 0.59 U/mL) was incubated with 50 µL of the compound or mixture (120 mg/L), followed by 50 µL each of CDNB and reduced glutathione (GSH). Kinetic absorbance was measured at 340 nm for 8 min. Blanks (without substrate) and quercetin (positive control) were included. *Inhibition* (%) was calculated using Equation (5), and treatment differences were evaluated via parametric ANOVA (α = 0.05) in GraphPad Prism 8.0.2 [42,43]. Each experiment was performed independently three times, with three technical replicates per experiment.

(5) $$\%\;Inhibition=1-\left(\frac{A_{m}-A_{B}}{A_{C}\;}\right)\times 100$$

where $A_{m}$ is the absorbance of the sample, $A_{B}$ is the absorbance of the blank, and $A_{C}$ is the absorbance of the negative control.

#### 2.4.4. CAT Activity Assay

Sinha’s method was adapted [48]. In Eppendorf tubes, 100 µL of protein extract (24.77 U/mL) was mixed with 100 µL of the compound or mixture (120 mg/L) and incubated at 37 °C for 15 min. Then, 200 µL of H_2_O_2_ was added and shaken for 2.5 min. The reaction was stopped by adding 600 µL of K_2_Cr_2_O_7_, followed by incubation at 100 °C for 10 min. A 200 µL aliquot was transferred to a 96-well plate, and absorbance was recorded at 570 nm [49]. Blanks and sodium azide (positive control) were used. The percentage of inhibition was calculated using Equation (5). Differences among treatments were analyzed via ANOVA (α = 0.05) in GraphPad Prism. Each experiment was performed independently three times, with three technical replicates per experiment.

#### 2.4.5. AChE Activity Assay

A modified Ellman method was used [50,51]. Protein extract (50 µL; 0.31 U/mL) was incubated with 50 µL of compound or mixture (120 mg/L) at 37 °C for 15 min. Then, 50 µL of DTNB and 50 µL of ATChI were added. Absorbance at 412 nm was monitored kinetically for 35 min [52]. Inhibition percentages were calculated using Equation (5). Mixtures causing ≥50% inhibition were reassessed at 15 mg/L. Differences and CI_50_ values were determined using ANOVA (α = 0.05) in GraphPad Prism. Each test included three replicates across three independent experiments, with blanks and dichlorvos (Nuvan^®^ 50 EC, Cenrogral SAS, Neiva, Colombia) as a positive control [53].

## 3. Results and Discussion

### 3.1. Insecticide Activity

#### 3.1.1. Fumigant Toxicity

**Figure 1:**
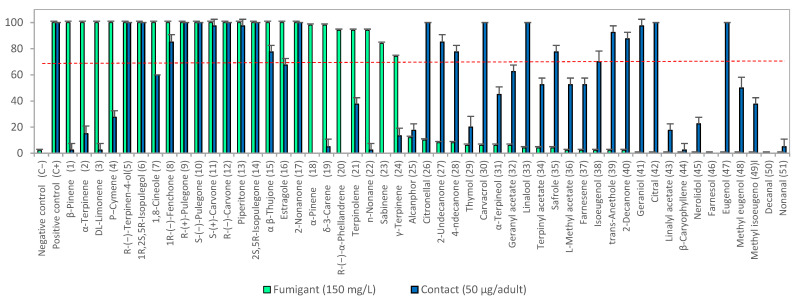
Preliminary screening of insecticidal activity of 51 plant-derived volatile compounds (VCs) against *S. zeamais*. Green bars represent fumigant activity at 150 mg/L. Blue bars represent contact toxicity at 50 µg/adult. Bars above the red line indicate ≥50% mortality, selected for LC_50_ or LD_50_ determination.

A total of 51 plant-derived volatile compounds (VCs) were screened for insecticidal activity, of which 24 showed fumigant toxicity at 150 mg/L (0.6–1.1 mM). These were labeled C1 to C24 (Figure 1). Among the active fumigant compounds, the majority were oxygenated monoterpenoids (13 VCs) and hydrocarbon monoterpenoids (9 VCs). This predominance is consistent with previous reports on the fumigant activity of monoterpenoids [54,55,56]. Notably, this study is the first to report the fumigant activity against *S. zeamais* of the following compounds: *1R*,*2S*,*5R*-isopulegol, citronellal, *2S*,*5R*-isopulegone, geranyl acetate, linalyl acetate, L-menthyl acetate, terpinyl acetate, farnesene, farnesol, isoeugenol, methyl isoeugenol, trans-anethole, 4-undecanone, decanal, and *n*-nonane.

Table 1 summarizes the fumigant and contact toxicity of compounds C1 to C37 across varying concentrations. Among these, C1–C7, C22, and C23 demonstrated the highest fumigant activity against *S. zeamais* (highlighted in green), with LC_50_ values below 30 µM and slope values above 0.50. These compounds produced steep dose–response curves, indicating that slight increases in concentration significantly enhanced lethality, comparable to the positive controls. This study provides the first LC_50_ estimates for fumigant toxicity against *S. zeamais* for *1R*,*2S*,*5R*-isopulegol (C7), *n*-nonane (C20), and *2S*,*5R*-isopulegone (C23). Compounds C4 (LC_50_: 1.42 mg/L) and C5 (LC_50_: 4.03 mg/L) exhibited toxicity levels like those reported in 7-day exposure assays (2.76 and 4.79 mg/L, respectively) [57,58], suggesting rapid fumigant action. Furthermore, the activity of compounds C12, C13, C14, and C19 appears to be driven primarily by inhalation exposure rather than contact, as their LC_50_ values align with previous reports in vapor-phase assays without physical barriers: 23.3 mg/L [59], 56.2 mg/L, 72.7 mg/L [60], and 120 µL/L [61], respectively.

**Figure 2:**
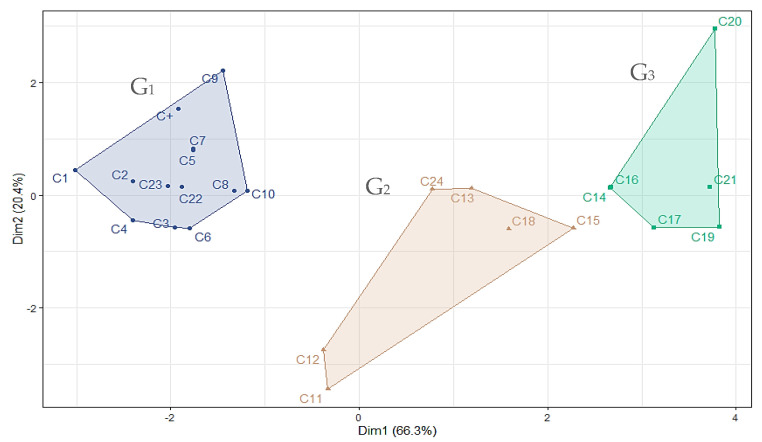
K-means cluster analysis of 24 plant-derived VCs (C1–C24), and a positive control (C+), based on LC_50_ values (fumigant toxicity) against *S. zeamais.* The optimal number of clusters (K = 3) was determined using Hubert and D indices: G1 = high toxicity, G2 = moderate toxicity, G3 = low toxicity.

The cluster analysis of fumigant-active VCs (Figure 2) identified three distinct groups. Cluster 1 (G1) includes the most potent compounds, with LC_50_ values below 13 mg/L and steep slopes. These are primarily monoterpenes with ketone and alcohol functionalities, which may enhance toxicity by forming hydrogen bonds with water molecules lining the insect tracheoles [62]. Cluster 2 (G2) contains compounds with moderate fumigant activity (LC_50_: 28.0–81.0 mg/L), including aromatic and hydrocarbon monoterpenes such as estragole (C11), a phenylpropanoid. Lower toxicity in structurally similar compounds like trans-anethole may result from double bond positioning near the aromatic ring [63]. Cluster 3 (G3) comprises compounds with low fumigant activity (LC_50_ > 88 mg/L and shallow slopes), mostly hydrocarbons. Their limited efficacy may be due to excessive lipophilicity, leading to accumulation on the insect cuticle and reduced internal uptake [62,64]. However, compounds with higher vapor pressures may still achieve moderate efficacy due to enhanced volatility and accessibility [62].

**Figure 3:**
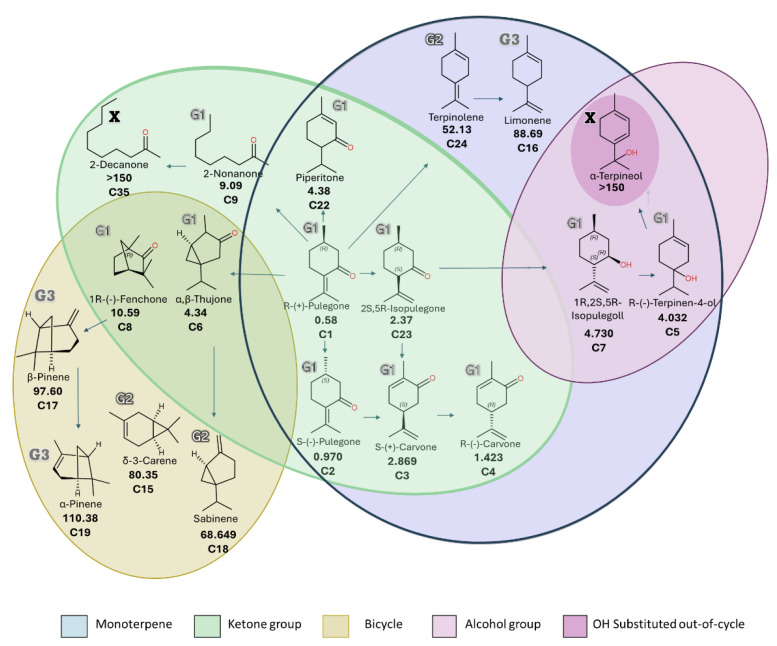
Venn diagram of LC_50_ values (ppm) for selected volatile compounds with fumigant activity against *S. zeamais*, grouped by carbon chain, compound type, and functional groups. “X” indicates compounds with <60% mortality at 150 ppm.

The preliminary structure–activity relationship (SAR) analysis revealed that a monocyclic monoterpenoid scaffold was common to 13 of the 24 active compounds. Among monocyclic hydrocarbon monoterpenes (C13, C14, C16, C21, and C24), fumigant activity was generally low. The addition of a hydroxyl group to the ring, as in C5 and C7 (including α-terpineol), increased LC_50_ values by approximately 18-fold. In contrast, ketone substitution enhanced toxicity by ~38-fold, as observed in C1–C4, C22, and C23. Bicyclic monoterpenes bearing ketone groups (C6 and C8) were, on average, 13.3 times more toxic than their hydrocarbon counterparts (C15, C17, C18, and C19), likely due to their higher polarity and volatility (Figure 3). These findings align with QSAR models showing rapid fumigant action in pests exposed to oxygenated hexacyclic structures [62,64,65].

Ketone-containing compounds were generally more toxic, with potency decreasing as the carbon chain length increased among non-terpenoid precursors. For example, 2-nonanone (C9) showed lower activity than shorter-chain analogs, and activity further declined with longer chains such as 2-decanone (C35). Bicyclic compounds derived from the pinyl cation were about twice as toxic as those derived from the terpinen-4-yl cation, as observed in the comparison of C6 vs. C8 and C18 vs. C17 and C19. These trends likely result from lower vapor pressures in larger molecules, which reduce volatility and bioavailability. Increased lipophilicity may also limit insect uptake [66] Additionally, greater unsaturation (particularly exocyclic double bonds) in monocyclic hydrocarbons and oxygenated monoterpenes was associated with enhanced fumigant toxicity. For example, C24 (with an exocyclic double bond) was about twice as potent as C16. Similarly, C1 and C2 were 3.7 times more toxic than structurally related compounds C3, C4, and C22. Stereochemistry also influenced activity: monoterpene ketones with the *R*-enantiomer configuration (C1 and C4) were approximately twice as toxic via inhalation as their *S*-enantiomers (C2 and C3). This enantioselectivity has been linked to more effective acetylcholinesterase (AChE) inhibition in stored-product pests. For instance, (R)-linalool binds more efficiently to the active site of AChE than its (S)-form [67].

Compounds that did not demonstrate significant fumigant activity at 150 mg/L included aldehydes, long-chain aliphatic ketones (with more than 10 carbons), sesquiterpenoids, and most phenylpropanoids (except for estragole). Monoterpenoids bearing acetate, phenol, or hydroxyl groups outside the ring system also showed poor activity. This includes alcohols such as linalool and α-terpineol, which are less effective fumigants, likely due to unfavorable molecular geometry that limits interaction with octopaminergic receptors, as compared to other oxygenated monoterpenes [68].

#### 3.1.2. Contact Toxicity

Preliminary screening identified 26 of the 51 volatile compounds (VCs) evaluated as active via contact toxicity at 50 µg/adult (Figure 1). These included 17 monoterpenes, 5 phenylpropanoids, and 4 aliphatic ketones, mainly coded between C1 and C37. This study reports, for the first time, the contact toxicity against *S. zeamais* of the following compounds: phellandrene, γ-terpinene, terpinolene, thymol, carvacrol, *1R*,*2S*,*5R*-isopulegol, *2S*,*5R*-isopulegone, farnesene, farnesol, methyl isoeugenol, trans-anethole, 2-nonanone, 2-decanone, 2-undecanone, and 4-undecanone. These results are consistent with previous findings in the literature [55,69,70,71].

According to the Probit analysis (Table 1), compounds C1, C2, C22, C23, and C25 demonstrated the most potent contact insecticidal activity, with LD_50_ values below 12 µg/adult and slope values greater than 0.26. These steep slopes indicate that small increases in dose significantly increase lethality, making these compounds comparable to the positive control. Additionally, this study reports, for the first time, 24-h LD_50_ values for compounds C4 (16.89 µg/adult), C20 (21.42 µg/adult), C29 (26.25 µg/adult), and C33 (36.94 µg/adult), which exceed values previously reported for 7-day assays (2.79, 17.62, 13.9, and 8.54 µg/adult, respectively) [58,72]. This suggests that the toxic effects of these compounds persist beyond the initial 24-h period.

**Figure 4:**
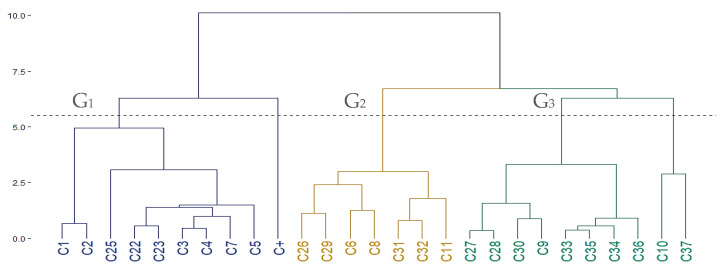
Cluster analysis of 26 plant-derived volatile compounds (C1–C 36), and a positive control (C+) based on contact toxicity against *S. zeamais*. Compounds were grouped using the hierarchical AGNES method. The optimal number of clusters (K = 3) was determined using Hubert and D indices; dotted lines indicate cluster separation: Group 1 (G1) = high toxicity, Group 2 (G2) = moderate to low toxicity, and Group 3 (G3) = low toxicity.

The cluster analysis of contact toxicity (Figure 4) revealed three groups. Cluster 1 (G1) included highly active monoterpenoids containing ketone or alcohol groups, with LD_50_ values below 20 µg/adult. These compounds exhibited low partition coefficients, indicating reduced lipophilicity enhances knockdown effects. Clusters 2 (G2) and 3 (G3) showed overlapping levels of moderate activity. G2 primarily included bicyclic monoterpenic ketones and phenylpropanoids with LD_50_ values ranging from 20 to 46 µg/adult and was characterized by greater unsaturation in the carbon skeleton. G3 comprised moderately to mildly active compounds (LD_50_: 21–73 µg/adult), including acyclic oxygenates and 1,8-cineole. The reduced efficacy of these compounds on *S. zeamais* is likely related to higher LogKow values, which promote retention in the insect cuticle and limit bioavailability, consistent with prior findings [62,64].

**Figure 5:**
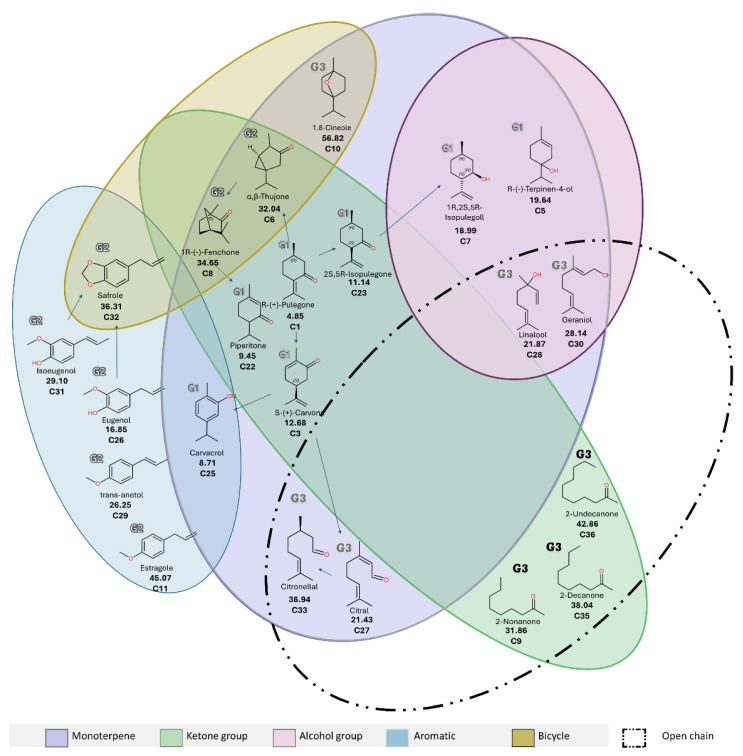
Venn diagram of LD_50_ values (µg/adult) for selected volatile compounds with contact toxicity against *S. zeamais*, grouped by carbon chain, compound type, and functional groups.

The preliminary structure–activity relationship (SAR) analysis supported the role of oxygenated monoterpenoids in mediating contact toxicity. Of the 26 active compounds, 17 were oxygenated monoterpenes. Monocyclic monoterpenic ketones (C1–C4, C22, and C23) were approximately 3.5 times more potent than aliphatic (C34–C36) and bicyclic (C6, C8, and C9) analogs (Figure 5). Increasing the carbon chain length in aliphatic ketones (C9, C35, and C36) was associated with reduced insecticidal activity. Monoterpenoids with alcohol groups (C28 and C30) showed 1.3 times higher efficacy when arranged in monocyclic structures (C5 and C7). Conversely, aldehydes (C27 and C33) displayed low activity, likely due to their acyclic nature. These results are consistent with previous reports showing that short-chain, monocyclic alcohols have greater contact and knockdown activity than their long-chain or acyclic counterparts [73,74].

The evaluation of ether-containing heterocycles revealed that C10 was approximately twice as inactive as other oxygenated bicyclic monoterpenes (C6 and C8), and the phenylpropanoid C32 was 1.5 times less active than its biosynthetic precursors C26 and C31. These observations suggest that oxygen-containing heterocyclic structures may reduce contact toxicity against *S. zeamais*. No significant differences in toxicity were observed between *cis*- and *trans*-stereoisomers of phenylpropanoids or among their hydroxy- (C31 and C26) and methoxy-substituted analogs.

The relatively low contact toxicity and moderate fumigant activity of hydrocarbon-type compounds may be attributed to their higher lipophilicity, which promotes cuticular retention and limits systemic penetration [62,64]. In contrast, the most active insecticidal group (G1), encompassing compounds with both fumigant and contact effects (C1–C10, C22, C23, and C25), consists mainly of monocyclic oxygenated monoterpenes. Within this group, the presence of an α,β-unsaturated exocyclic ketone emerged as a key structural feature associated with potent dual insecticidal activity [28,54,62].

### 3.2. Design of Mixtures of Bioactive Volatile Metabolites and Their Insecticide Effect

#### 3.2.1. Mixture Pre-Design and RSM Modeling

Mixtures were pre-designed based on the structural features of plant-derived volatile compounds (VCs) identified through cluster analysis (Table 2). The mixtures were pre-designed based on the structural characteristics of plant-derived volatile compounds (VCs), as determined by cluster analysis (Table 2). A total of 20 mixtures for fumigant toxicity (M1–M20) and 9 for contact toxicity (MC1–MC9) were formulated and evaluated using the response surface methodology (RSM) at doses below the LC_90_ or LD_90_ of the most active component. A second-order polynomial model was applied, yielding statistically significant results (*p* < 0.001; F-statistic > 10.0). The model demonstrated strong predictive power, with R^2^ values > 0.850 and differences between R^2^, adjusted R^2^, and predicted R^2^ below 0.10, supporting its reliability through ANOVA (Tables S8 and S9). Most ternary mixtures significantly rejected the null hypothesis (H_0_: μ_i_ = 0) across all pre-designed 50:50 combinations in the individual ANOVAs, except for C22 + C16 in MC6, C28 + C29 in MC8, C10 + C19 in M14, and C1 + C22 in M18, which were excluded from the model due to non-significant effects. Additionally, C25 + C26 in MC1 exhibited antagonism (coefficient = −1.60). These exclusions enabled the prediction of secondary mixtures, which in most cases provided the best responses [75].

#### 3.2.2. Toxicity Effect of Predicted Mixtures by RMS

**Figure 6:**
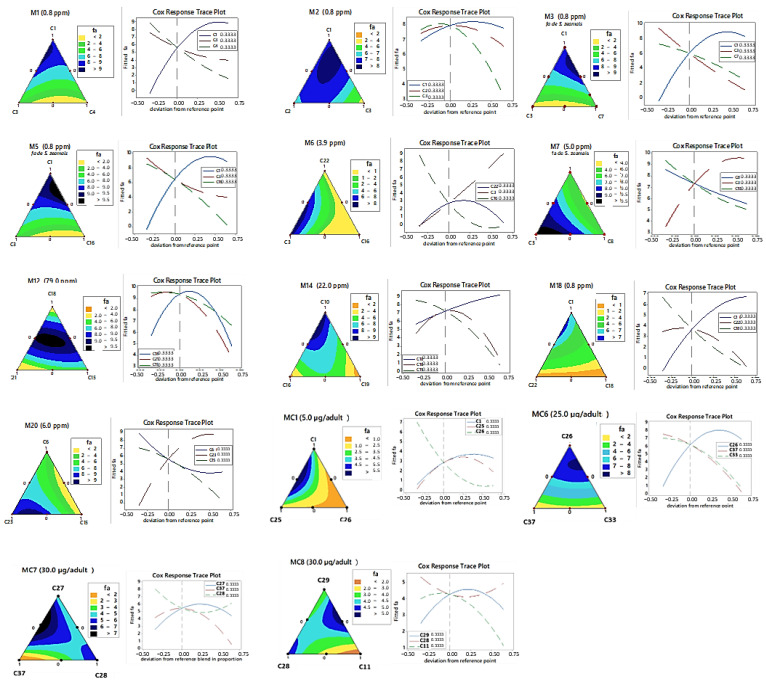
Response surface plots (RSM) and Cox effect traces showing the optimized insecticidal activity of volatile compound mixtures against *S. zeamais*. Contour plots represent the centroid estimated by the quadratic model in a {3.2} simplex lattice design. Ten fumigant mixtures (M1–M3, M5–M7, M12, M14, M18, and M20) and five contact mixtures (MC1 and MC6–MC9) are shown, optimized for maximum efficacy.

RSM estimated ten fumigant mixtures with insecticidal potential: two ternary mixtures (M2 and M12) and eight binary mixtures (M1, M3–M7, M14, M16, M18, and M20), as well as five mixtures with knockdown/contact effects, including four binary (MC1 and MC6–MC8) and one ternary (MC9) mixture. These are listed in Table 2 under the “component ratio” column. Response surface plots (Figure 6) visualized insecticidal efficacy, with the affected fraction (fa) ranging from high (black) to low (orange). Cox effect trace plots highlighted the contribution of individual components, particularly R-(+)-pulegone (C1), which showed consistently positive slopes and prominent activity across both modes of action (Figure 6, M1–M3, M12 and MC6). Similar efficacy patterns were observed for C3 in M6 and M7, C10 in M14, C23 in M20, and C26 in MC6. The ternary mixture M12 displayed strong fumigant activity, attributed to synergism between sabinene (C18—α-terpinen-4-yl cation) and δ-3-carene (C15—α-terpinyl cation). In contrast, mixtures lacking these components (e.g., M10 and M11) exhibited antagonistic fumigant effects, underscoring the critical role of C18 and C15 in enhancing toxicity.

Each mixture was tested against *S. zeamais* at the LC_50_ or LD_50_ of its most active component. As shown in Table 2, six fumigant mixtures (M1, M2, M3, M5, M12, and M20) and three contact toxicity mixtures (MC1, MC6, and MC8) achieved ≥50% mortality. An additional mixture, M21, a racemic blend of pulegone (C1 + C2), was also included, bringing the total to ten. These mixtures were further evaluated to assess dose–response behavior and component interactions under both exposure routes.

Table 3 presents comparative insecticidal data. Eight out of ten mixtures demonstrated significant insecticidal activity via both fumigant and contact routes. M2 (C1 + C2 + C3) was the most effective, with an LC_50_ of 0.48 mg/L and an LD_50_ of 6.36 µg/adult. In contrast, MC6 (LD_50_: 18.33 µg/adult) showed no fumigant activity, while M12 (LC_50_: 39.22 mg/L) was ineffective in contact toxicity. Notably, the LC_50_ values for M1, M2, M3, M5, and M21 were lower than that of the positive control dichlorvos (2.17 mg/L). All mixtures, except M12, MC6, and MC8, showed insecticidal potency comparable to the controls, including cypermethrin (LD_50_: 10.49 µg/adult).

#### 3.2.3. Interaction Analysis: Synergism and Antagonism

All tested combinations conformed to the median-effect mass-action model (R ≥ 0.97), allowing the calculation of CI and DRI values (Table 3). CI and log DRI plots across four lethality levels (30%, 50%, 75%, and 90%) are shown in Supplementary Figure S7. Antagonistic interactions (CI > 1.2) were detected in six knockdown mixtures, MC1 in the fumigant model and M1, M5, M21, MC1, and MC8 in the contact toxicity model. In contrast, MC6 displayed moderate synergism (CI = 0.76) with favorable dose reduction indices (DRIs) across all effect levels (Figure S7), while M2 and M20 showed additive interactions (CI ≈ 1.09; DRI ≈ 1.10) [40].

Six of the seven fumigant-active mixtures showed synergistic interactions (CI < 1). M12 and M2 exhibited strong synergism (CI = 0.54 and 0.64, respectively). Despite its focus on contact toxicity, MC8 also showed mild synergism. Fumigant mixtures generally had DRI values > 1, indicating that lower doses of individual components could be used in combination. Interestingly, components present in lower proportions (<0.40) often had higher DRIs than their majority counterparts, suggesting that the minor component may act unimpeded. This aligns with previous studies indicating that insects preferentially metabolize the more abundant terpene in a mixture, allowing the minor component to exert a more potent toxic effect. For example, the racemic mixture M21 (equal parts C1 and C2) demonstrated additive interaction (CI = 0.91) and balanced DRI values (1.77 and 2.90), supporting this hypothesis. This behavior is consistent with prior work showing that pulegone–citronellal mixtures enhanced toxicity by inhibiting cytochrome P450 enzymes in *Musca domestica* (Diptera Muscidae) [76].

Despite its high efficacy (LC_50_: 1.56 mg/L), MC1 exhibited antagonism (CI_50_ = 2.19), likely due to the low DRI of R-(+)-pulegone (C1; DRI = 0.45) at higher concentrations. In contrast, carvacrol (C25) showed an exceptionally high DRI (732.7), significantly boosting the fumigant activity of the mixture. This interaction is both biologically and economically advantageous, as C1 is approximately 10 times more expensive than C25 (Sigma-Aldrich^®^, Saint Louis, MO, USA). Notably, both compounds are approved flavoring agents (FEMA Nos. 2963 and 2245) with known safety profiles: oral LD_50_ for carvacrol = 2462.23 mg/kg and for R-(+)-pulegone = 810 mg/kg (oral), 80 mg/kg (i.v.), and 73 mg/kg (i.p.) [77,78]. This synergistic interaction between carvacrol and a monoterpene ketone has previously been observed in studies on the fumigant activity against *Culex quinquefasciatus* [79].

Similarly, D,L-limonene (C16) in mixture M5 had the second-highest DRI (511.1), indicating strong synergism. The availability of limonene from citrus waste further enhances its practical value. Among all tested mixtures, M1, M2, M3, and M20 emerged as the most potent insecticides, demonstrating strong efficacy across both exposure routes and marked synergism in fumigant activity. The racemic mixture M21 also exhibited additive fumigant effects without requiring enantiomeric separation (Figures S7 and S8c).

Among all the tested combinations, M1, M2, M3, and M20 were the most effective, showing strong fumigant and contact activity and synergism in the fumigant model. These mixtures caused >50% mortality within 24 h, with LC_50_ values ranging from 0.54 to 0.63 mg/L and LD_50_ values between 6.36 and 9.17 µg/adult. In comparison, literature reports indicate that deltamethrin (1 ppm) caused only 45% mortality after 14 days and permethrin–methyl (4 ppm) achieved 100% mortality in 72 h [79,80]. The natural mixtures evaluated in this study demonstrated comparable or superior efficacy at lower doses and with shorter exposure times, underscoring their potential as environmentally friendly alternatives. Furthermore, the reported LD_50_ of permethrin (42.75 µg/adult) was substantially higher than that of most mixtures tested in our work [81,82].

### 3.3. Effects of the Most Active Mixtures and Their Components on the Nervous System and Detoxifying Enzymes in S. zeamais

Three independent protein extracts from *S. zeamais* were prepared and characterized to assess acetylcholinesterase (AChE), catalase (CAT), and glutathione S-transferase (GST) activity. Enzymatic activity and assay conditions were optimized and are summarized in Table S10.

#### 3.3.1. Inhibitory Effects on Acetylcholinesterase (AChE)

**Figure 7:**
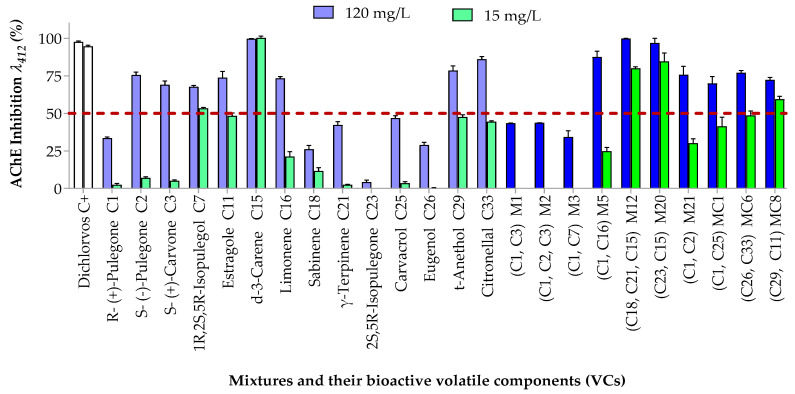
AChE inhibition in *S. zeamais* by mixtures and individual components at 120 and 15 mg/L is shown (mixtures: blue/green; components: light blue/light green). Bars above the red line at 120 mg/L indicate ≥50% inhibition, selected for IC_50_ analysis. Error bars represent SD from three independent experiments.

Figure 7 shows the inhibitory activity of mixtures and their constituents on AChE activity in *S. zeamais*. At 120 mg/L, eight volatile compounds (VCs) significantly inhibited AChE by more than 50% (light blue bars), with significant differences among treatments (*p* < 0.001, *F* = 78.72, R^2^ = 0.948). These same compounds were then evaluated at 15 mg/L, a concentration chosen as intermediate between the LC_50_ values of G1 and G2 clusters identified in the fumigant assay. Again, significant differences were observed (*p* < 0.001, *F* = 134.0, R^2^ = 0.973). Among the tested compounds, δ-3-carene (C15) showed the strongest inhibitory effect, comparable to that of the positive control. This study is the first to report AChE inhibition in *S. zeamais* by compounds C7, C11, C15, C16, C21, C23, C26, and C33 and the enantiomers C1 and C2. Carvacrol (C25), which inhibited AChE by only 46.85%, was excluded from further testing. This is consistent with previous studies reporting an IC_50_ of 19.4 µM (~118 mg/L) for carvacrol against *S. zeamais* AChE [52].

IC_50_ values were subsequently determined for the eight compounds with strong inhibitory potential (Table 4 and Figure S9). Among these, C15 (IC_50_ = 80.35 mg/L) and C29 (IC_50_ = 4.24 mg/L) were the most potent. Monoterpenes derived from α-terpinyl cation (C15 and C16) were approximately 20% more inhibitory than those from α-terpinen-4-yl cation (C18 and C21). Phenylpropanoids derived from coumaryl acetate (C11 and C29) showed 2.6-fold higher inhibition than those from coniferyl acetate (C26). *t*-Anethole (C29) was 6.5 times more potent than its isomer estragole (C11), likely due to extended conjugation in its aromatic ring. This difference is attributed to the extended conjugation of the aromatic ring in C29, which likely enhances its electronic interactions with the enzyme’s active site.

Oxygenated monoterpenoids (C2, C3, and C7; IC_50_ = 36.60, 66.27, and 18.15 mg/L, respectively) also displayed relevant neurotoxic potential. These results align with QSAR models showing that the orbital electronegativity of carbonyl groups facilitates Michael-type additions with electron-rich enzymatic sites [54]. Dipole interactions and Van der Waals forces further stabilize binding to AChE. Stereoselectivity was observed: the *S*-enantiomer of pulegone (C2) showed 2.3 times higher inhibition than the *R*-enantiomer (C1). α,β-Unsaturated ketones (C1–C3) had 14-fold greater inhibitory activity than saturated ketones like C23. This finding is consistent with previous insecticidal studies on *S. zeamais*, which have demonstrated that the presence of a double bond between the α and β carbons adjacent to a carbonyl group increases the polarizability of the molecule. This enhanced polarizability contributes to stronger intermolecular interactions, allowing the compound to bind more effectively to proteins and nucleic acid targets. As a result, it can disrupt key physiological and metabolic processes in the insect [54,80].

Seven of ten tested insecticidal mixtures reduced AChE activity by more than 50% (Figure 7, blue bars). These were further tested at 15 mg/L, with M12 and M20 exceeding 80% inhibition at both concentrations. Despite C1’s strong insecticidal activity, its neurotoxicity was moderate (33.52%), both as a pure compound and in mixtures such as M1 (43.49%), M2 (43.67%), and M3 (34.18%), indicating that AChE inhibition is not its primary mode of action. IC_50_ values for the seven mixtures (Table 4) revealed M12 (IC_50_ = 0.81 mg/L) and M20 (IC_50_ = 0.61 mg/L) as the most potent. Lineweaver–Burk plots (Figure S10) identified competitive inhibition for all active oxygenated compounds (C2, C3, C7, C11, C29, and C33) and their mixtures. Even mixtures with limited AChE inhibition, such as M5 (R-(+)-pulegone + DL-limonene), followed this inhibition type. These findings, aligned with previous literature, suggest that monoterpenoids with organic ketone and alcohol function act as competitive inhibitors of AChE catalytic activity in other insect species [13,54,81]. In contrast, hydrocarbon-type monoterpenes (C15 and C16) and mixtures composed solely of them (M21) exhibited non-competitive inhibition, suggesting binding at allosteric sites or at the entrance to the enzyme’s active site. These compounds may also interact with the thiol group of cysteine residues, a key feature in many enzyme active sites [13,82]. The high efficacy of C15 in the neurotoxicity assay may be due to steric interactions from its bicyclic structure.

The AChE-inhibiting mixtures were further analyzed to evaluate synergistic effects. Combination indices (CIs) and dose reduction indices (DRIs) were calculated for a 50% inhibitory effect (Table 5). CI and log DRI plots were generated at 15 mg/L, 120 mg/L, and LC_50_ levels (Figure S9). All combinations adhered to the law of mass action (r ≥ 0.940). Synergistic interactions were observed in five of the seven mixtures. Despite their high AChE inhibition, M12 (CI = 1.95) and M20 (CI = 2.31) showed antagonism (Figure S11-2,11-3), likely due to unfavorable DRI values for C15. In M20, the combination with C23 may be underutilized for this target. This is supported by the very high DRI of C23 (42,279.60) and the overall strong efficacy of M20 across toxicity modes. These results align with existing literature suggesting that monoterpenes can affect multiple targets in insects, with numerous mechanisms of toxicity [63].

Mixtures like MC1 (R-pulegone + carvacrol, IC_50_ = 22.32 mg/L) and MC6 (eugenol + citronellal, IC_50_ = 4.45 mg/L) showed strong synergism. The interaction between carbonyl and hydroxyl groups may enhance binding affinity for AChE. QSAR data support this, suggesting that higher orbital electronegativity and extended conjugation increase the likelihood of Michael addition with the enzyme [25,53]. However, some mixtures combining monoterpenoids with ketone and alcohol groups, such as M1, M2, and M3, showed antagonism, likely due to steric hindrance involving the R-pulegone enantiomer (C1).

#### 3.3.2. Inhibitory Effects on Catalase (CAT) Activity

**Figure 8:**
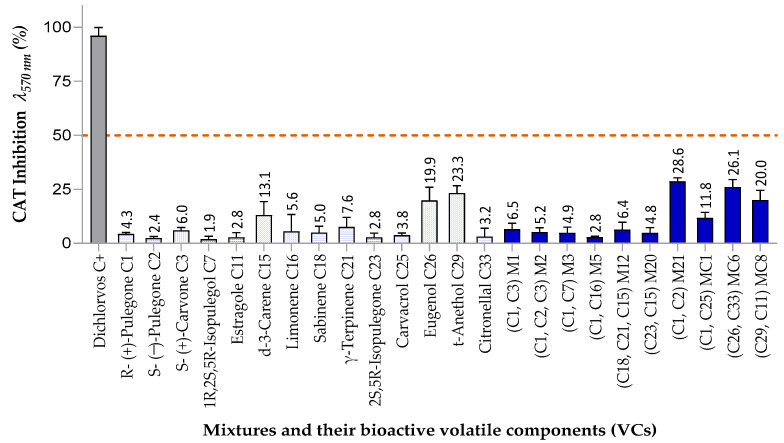
Inhibitory effect of mixtures and individual components on catalase (CAT) activity in *S. zeamais* at 120. mg/L Mixtures (bright blue bars) and their components (light blue bars) Bars above the red line at 120 mg/L indicate ≥50% inhibition, selected for IC_50_ analysis. Error bars represent SD from three independent experiments.

Figure 8 shows the inhibitory effects of mixtures and their components on CAT activity in *S. zeamais* at 120 mg/L. Significant differences were observed among treatments (*p* < 0.001, *F* = 50.81, R^2^ = 0.868). This study is the first to explore the effect of these compounds on oxidative stress enzymes in this species. At this concentration, inhibition did not exceed 43.7%. Two oxygenated phenylpropanoids, C26 (19.9%) and C29 (23.3%), showed moderate activity, aligning with previous findings on compounds such as menthone, which reduced CAT activity by ~30% in *S. oryzae* [83].

C29 inhibition was eight times greater than that of its isomer C11, which correlates with their AChE inhibition profiles, suggesting that terminal unsaturation in phenylpropanoids may reduce CAT detoxification and increase neurotoxic effects. Mixtures M21, MC6, and MC8 exhibited moderate CAT inhibition (20–29%), primarily attributed to their phenylpropanoid components (C26 and C29). These effects were like those of the individual compounds, indicating additive interactions. Notably, M21 (racemic pulegone) showed six times more CAT inhibition (28.6%) than its components C1 (4.3%) and C2 (2.4%), highlighting the potential of enantiomeric mixtures to enhance bioactivity and reduce costs. CAT inhibition likely leads to peroxide accumulation and oxidative damage, particularly in nervous tissues, which may explain the knockdown effect observed in some mixtures [84,85].

#### 3.3.3. Inhibitory Effects on Glutathione S-Transferase (GST) Activity

**Figure 9:**
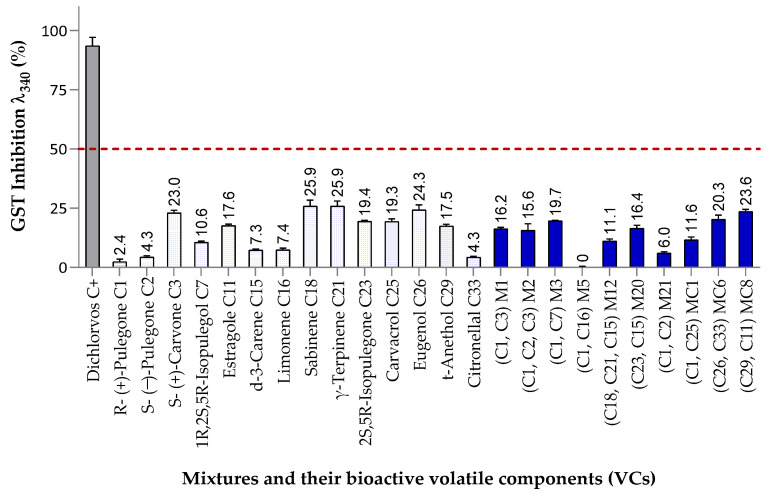
Inhibitory effect of mixtures and individual components on glutathione S-transferase (GST) activity in *S. zeamais* at 120 mg/L. Mixtures (bright blue bars) and their components (light blue bars) Bars above the red line at 120 mg/L indicate ≥50% inhibition, selected for IC_50_ analysis. Error bars represent SD from three independent experiments.

Figure 9 presents the effects of VCs on GST activity in S. zeamais at 120 mg/L. This is the first report evaluating these specific compounds on GST function in this species, showing significant differences between treatments (*p* < 0.001, *F* = 89.78, R^2^ = 0.929). Five compounds moderately reduced GST activity (19–26%). C3 and C23 had 2.5 times greater inhibition than C1 and C2, suggesting that exocyclic conjugation with a carbonyl group enhances GST inhibition. C11 and C29 had similar effects (~17.5%), indicating that the double bond position in phenylpropanoids does not significantly influence GST activity. No clear correlation was observed between GST inhibition and mode of insecticidal action: compounds toxic via inhalation (C18 and C21) and contact (C26 and C29) both showed moderate effects. Mixtures M3 (19.7%), MC6 (20.3%), and MC8 (23.6%) slightly reduced GST activity, mainly due to their phenylpropanoid constituents. However, their effects did not surpass those of the individual components, suggesting no synergistic enhancement. In some cases, GST inhibition was lower in mixtures than in individual compounds, possibly due to antagonistic interactions.

## 4. Conclusions

This study demonstrates the strong potential of plant-derived volatile compounds (VCs) as effective bioinsecticides against *S. zeamais*, particularly oxygenated monoterpenes bearing ketone and alcohol functional groups. These compounds exhibited notable insecticidal activity via both contact and fumigant exposure routes. Among them, pulegone enantiomers showed outstanding efficacy, outperforming commercial insecticides, such as dichlorvos and cypermethrin. The results also highlight the value of synergistic mixtures in enhancing insecticidal performance. Specifically, mixtures M2 (R-(+)-pulegone, S-(−)-pulegone, R-(−)-carvone) and M20 (isopulegone, δ-3-carene) demonstrated strong fumigant effects and neurotoxicity, primarily through acetylcholinesterase (AChE) inhibition. These mixtures also showed mild inhibitory effects on glutathione S-transferase (GST), supporting the presence of a multitarget mechanism of action. Such multitarget interactions may limit detoxification and delay resistance development, offering a promising strategy for sustainable pest control. Although GST and CAT are not the primary toxicological targets, their inhibition could contribute to reduced metabolic defense in insects. Overall, these findings reinforce the potential of plant-synthesized bioinsecticides as environmentally friendly alternatives to synthetic chemical insecticides.

## Figures and Tables

**Table 1:** Fumigant and contact toxicity of EOs components against *S. zeamais* at 24 h after exposure.

C	Compound	Fumigant Toxicity					Contact Toxicity		
		LC_50_ ^a^ (95% FL)		LC_90_ ^b^ (95% FL)		$\beta_{i}$ ^c^ (±SD)	LD_50_ ^d^ (95% FL)	LD_90_ ^e^ (95% FL)	$\beta_{i}$ ^c^ (±SD)
		mg L^−1^	µmol L^−1^	mg L^−1^	µmol L^−1^		µg/Adult	µg/Adult	
C1	*R*-(+)-Pulegone **^a^**	0.58 (0.46–0.71)	3.81 (3.03–4.69)	0.92 (0.77–1.26)	6.07 (5.09–8.25)	3.73 (±0.84)	4.85 (4.35–5.32)	7.40 (6.79–8.28)	0.51 (±0.060)
C2	*S*-(−)-Pulegone **^a^**	0.97 (0.69–1.21)	6.37 (4.54–7.98)	1.68 (1.39–2.37)	11.06 (9.17–15.56)	1.79 (±0.44)	7.44 (7.00–7.89)	9.64 (9.05–10.54)	0.58 (±0.075)
C3	*S*-(+)-Carvone **^a^**	2.87 (1.99–3.75)	19.10 (13.26–24.99)	5.31 (4.26–8.25)	35.36 (28.36–54.95)	0.52 (±0.14)	12.68 (10.91–14.60)	23.236 (20.46–27.42)	0.12 (±0.015)
C4	*R*-(−)-Carvone **^a^**	1.42 (1.14–1.72)	9.48 (7.57–11.48)	2.17 (1.84–2.88)	14.42 (12.23–19.20)	1.73 (±0.40)	16.89 (14.56–19.35)	28.45 (24.99–34.24)	0.11 (±0.012)
C5	*R*-(−)-Terpinen-4-ol **^a^**	4.03 (3.27–4.91)	26.14 (21.22–31.82)	6.13 (5.18–8.46)	39.76 (33.56–54.87)	0.61 (±0.15)	19.64 (18.42–21.45)	24.82 (22.64–29.63)	0.25 (±0.035)
C6	α, β-Thujone (70:10)	4.34 (3.49–5.30)	28.54 (22.90–34.79)	6.81 (5.75–8.94)	44.70 (37.75–58.73)	0.52 (±0.11)	32.04 (29.44–34.42)	44.67 (41.56–49.34)	0.10 (±0.013)
C7	*1R*,*2S*,*5R*-Isopulegol	4.73 (3.86–5.68)	30.66 (25.05–36.84)	7.19 (6.13–9.43)	46.64 (39.75–61.17)	0.52 (±0.11)	18.99 (17.7–20.15)	26.65 (25.07–28.90)	0.17 (±0.019)
C8	*1R*-(−)-Fenchone **^a^**	10.59 (8.56–13.16)	69.59 (56.24–86.45)	17.06 (14.18–23.81)	112.05 (93.12–156.40)	0.20 (±0.046)	34.65 (32.19–37.08)	48.33 (45.12–52.78)	0.09 (±0.010)
C9	2-Nonanone	9.09 (7.49–11.04)	63.92 (52.63–77.64)	13.73 (11.61–18.91)	96.54 (81.59–132.94)	0.28 (±0.067)	31.86 (30.27–33.17)	39.69 (38.01–42.15)	0.16 (±0.021)
C10	1.8-Cineole **^a^**	12.96 (10.38–16.10)	84.04 (67.31–104.36)	21.68 (18.02–29.56)	140.57 (116.82–119.66)	0.15 (±0.031)	56.55 (53.14–60.22)	79.45 (73.78–87.99)	0.056 (±0.002)
C11	Estragole	30.46 (22.40–39.81)	205.56 (151.13–268.62)	57.45 (45.99–87.88)	387.67 (310.36–593.03)	0.05 (±0.012)	45.59 (38.53–53.40)	79.66 (68.62–99.73)	0.038 (±0.005)
C12	p-Cymene **^a^**	28.68 (23.08–35.59)	213.67 (171.95–256.12)	46.96 (39.01–65.39)	349.89 (290.68–487.22)	0.07 (±0.016)	-	-	-
C13	α-Terpinene **^a^**	60.24 (47.80–72.75)	442.21 (350.86–533.98)	96.19 (81.75–125.28)	706.07 (600.09–919.60)	0.04 (±0.007)	-	-	-
C14	*R*-(−)-α-Phellandrene **^a^**	88.87 (69.70–108.27)	652.36 (511.64–794.79)	142.53 (119.87–197.61)	1046.26 (879.93–1450.53)	0.02 (±0.006)	-	-	-
C15	δ-3-Carene **^a^**	80.35 (64.09–97.18)	589.79 (470.47–713.38)	135.83 (114.92–178.25)	997.04 (843.60–1308.47)	0.02 (±0.005)	-	-	-
C16	DL-Limonene (1:1)	88.69 (74.72–103.70)	651.06 (548.49–761.24)	136.14 (117.96–171.69)	999.33 (865.87–1260.27)	0.03 (±0.005)	-	-	-
C17	β-Pinene **^a^**	97.60 (77.15–116.76)	716.41 (566.36–857.12)	151.34 (129.39–198.56)	1.110.93 (949.79–1457.51)	0.02 (±0.005)	-	-	-
C18	Sabinene **^a^**	68.65 (56.89–79.75)	503.92 (417.59–585.44)	102.10 (89.08–128.61)	749.50 (653.90–944.04)	0.04 (±0.008)	-	-	-
C19	α-Pinene **^a^**	110.38 (90.76–130.60)	810.25 (666.22–958.70)	178.81 (152.92–235.051)	1312.53 (1122.51–1725.39)	0.02 (±0.004)	-	-	-
C20	n-Nonane	109.12 (86.72–127.88)	850.81 (676.19–997.02)	171.55 (147.92–228.44)	1337.49 (1153.25–1781.07)	0.02 (±0.005)	-	-	-
C21	γ-Terpinene **^a^**	107.95 (82.31–146.97)	792.42 (604.23–1078.84)	187.96 (148.32–224.83)	1379.72 (1088.75–2384.44)	0.02 (±0.005)	-	-	-
C22	Piperitone	4.38 (3.72–5.35)	28.77 (24.45–35.13)	6.19 (5.26–8.89)	40.67 (34.54–54.43)	0.70 (±0.19)	9.45 (8.61–10.41)	14.70 (13.25–16.96)	0.24 (±0.031)
C23	*2S*,*5R*-Isopulegone	2.37 (1.86–2.89)	15.57 (12.19–18.96)	3.88 (3.29–5.18)	25.46 (21.47–34.05)	0.85 (±0.19)	11.14 (9.64–12.49)	18.48 (16.56–21.64)	0.17 (±0.022)
C24	Terpinolene **^a^**	52.13 (34.83–72.33)	337.92 (225.81–468.91)	88.83 (69.69–154.50)	575.86 (451.79–1001.61)	0.035 (±0.012)			
C25	Carvacrol	-	-	-	-	-	8.71 (7.92–9.56)	13.45 (12.19–15.38)	0.27 (±0.033)
C26	Eugenol	-	-	-	-	-	20.9 (19.22–22.65)	30.82 (28.35–34.42)	0.13 (±0.015)
C27	Citral	-	-	-	-	-	21.42 (19.34–23.61)	32.68 (29.77–36.82)	0.11 (±0.013)
C28	Linalool	-	-	-	-	-	21.87 (19.09–24.23)	36.23 (33.20–40.64)	0.09 (±0.011)
C29	trans-Anethol	-	-	-	-	-	26.25 (22.85–29.37)	46.08 (41.56–52.95)	0.06 (±0.008)
C30	Geraniol	-	-	-	-	-	28.13 (25.70–30.49)	41.06 (37.98–45.41)	0.01 (±0.011)
C31	Isoeugenol	-	-	-	-	-	29.1 (24.40–34.21)	66.2 (57.28–80.01)	0.03 (±0.004)
C32	Safrole	-	-	-	-	-	36.31 (30.15–42.37)	83.04 (73.50–96.63)	0.03 (±0.003)
C33	Citronellal	-	-	-	-	-	36.94 (34.60–39.14)	50.64 (47.46–55.39)	0.09 (±0.012)
C34	4-Undecanone	-	-	-	-	-	37.05 (34.09–40.01)	53.95 (49.63–60.68)	0.08 (±0.010)
C35	2-Decanone	-	-	-	-	-	38.04 (35.29–40.84)	57.17 (52.97–63.12)	0.07 (±0.007)
C36	2-Undecanone	-	-	-	-	-	42.86 (40.60–45.30)	57.13 (53.43–62.83)	0.09 (±0.011)
C37	Geranyl acetate	-	-	-	-	-	73.00 (65.98–81.05)	126.06 (112.84–146.08)	0.02 (±0.003)
C+	Dichlorvos	2.17 (1.33–3.81)	9.84 (6.01–17.22)	4.57 (3.21–8.75)	20.68 (14.53–39.59)	0.51 (±0.15)	-	-	-
C+	Cypermethrin	-	-	-	-	-	10.492 (0.00–29.96)	35.79 (21.98–84.37)	0.08 (±0.019)

Values represent means with confidence intervals and slope coefficients. Fumigant and contact toxicity assays were performed in three independent experiments (*n* = 5 and *n* = 4, respectively). *p* < 0.05. Compounds with highest fumigant activity are highlighted in green. ^a^ LC_50_: concentration causing 50% mortality; ^b^ LC_90_: concentration causing 90% mortality; ^c^ slope of the mortality regression curve; ^d^ LD_50_: dose causing 50% mortality; and ^e^ LD_90_: dose causing 90% mortality.

**Table 2:** Predicted mixtures by RMS and their insecticidal efficacy evaluated at the LC_50_ or LD_50_ of the most potent component.

Fumigant Toxicity							Contact Toxicity						
Code	Pre-Designed Mixes			RSM Estimated Mixtures			Code	Pre-Designed Mixes			RSM Estimated Mixtures		
	(A + B + C)			Components (Ratio)	ppm mg/L ^a^	Mortality (%) ± SE		(A + B + C)			Components (Ratio)	µg/ Adult ^b^	Mortality (%) ± SE
M1	C1	C3	C4	C1: C3 0.73: 0.27	0.6	67.5 ± 5.0	MC1	C1	C25	C26	C1: C25 0.79: 0.21	5	73.3 ± 5.8
M2	C1	C2	C3	C1: C2: C3 0.65: 0.14: 0.21	0.6	87.5 ± 5.0	MC2	C9	C25	C26	C1 1	ND	
M3	C1	C3	C7	C1: C7 0.67: 0.33	0.6	52.5 ± 5.0	MC3	C1	C25	C34	C1: C25 0.79: 0.21	ND	
M4	C1	C3	C5	C1: C3 0.73: 0.27	ND		MC4	C25	C25	C34	C25 1	ND	
M5	C1	C3	C16	C1: C16 0.74: 0.26	0.6	60.0 ± 8.2	MC5	C1	C5	C10	C1: 1.00 (combiner C10) *	ND	
M6	C22	C3	C16	C3: C22 0.80: 0.20	2.9	35.0 ± 5.7	MC6	C26	C37	C33	C26: C33 0.70: 0.30	20	57.5 ± 9.6
M7	C8	C3	C10	C8: C3 0.1: 0.90	2.9	7.5 ± 5.0	MC7	C27	C37	C28	C27: C37 0.59: 0.41	22	37.5 ± 5.0
M8	C8	C7	C6	C9: 1.00 (all combinations) *	ND		MC8	C29	C11	C28	C29: C11 0.60: 0.40	22	50.0 ± 8.2
M9	C12	C9	C10	C9: 1.00	ND		MC9	C22	C10	C28	C10: C28: C22 0.65: 0.14: 0.21	9.5	40.0 ± 8.2
M10	C20	C19	C17	C17: 1.00	ND								
M11	C14	C19	C16	C16: 1.00	ND								
M12	C18	C21	C15	C18: C21: C15 0.46: 0.31: 0.23	66.4	97.0 ± 5.8							
M13	C14	C12	C16	C12: 1.00	ND								
M14	C10	C16	C19	C10: C16 0.75: 0.25	13	7.5 ± 5.0							
M15	C11	C12	C13	C11: 1.00 (all combinations) *	ND								
M16	C10	C16	C18	C10: C16 0.75: 0.25	ND								
M17	C10	C5	C18	C5:1.00 (combiner C5) *	ND								
M18	C1	C22	C18	C1: C22 0.75: 0.25	0.6	40.0 ± 5.0							
M19	C3	C15	C18	C3: 1.00	ND								
M20	C6	C23	C15	C23:C15 0.74: 0.26	2.9	70.0 ± 8.2							

^a^ LC_50_-based evaluation concentration of the most active component; ^b^ LD_50_-based evaluation dose of the most active component; * indicates mixtures with antagonistic effects in one or more combinations; and ND: not determined due to model rejection or lack of a valid mixture prediction.

**Table 3:** Insecticidal effect and interaction of plant-synthesized volatile compounds (VCs) against *S. zeamais*, based on the median effect of the law of mass action.

Mixes A (Ratio) B (Ratio) C (Ratio)	Fumigant Toxicity					Contact Toxicity				
	${LC}_{50i}$ ^a^ (LC-95%) mg/L	$\beta_{i}$^b^ ± SD	DRI ^c^	CI_50_ ^d^	Interaction	${LD}_{50i}$ ^e^ (LC-95%) µg/Adult	$\beta_{i}$ ^b^ ± SD	DRI ^a^	CI_50_ ^d^	Interaction
**M1** C1 (0.73) C3 (0.27)	**0.54** **(0.38–0.73)** 0.58 2.87	2.54 ± 0.78	1.41 18.1	0.77	Moderate synergism	**9.17** **(4.09–13.97)** 4.85 12.68	0.16 ± 0.60	0.70 4.77	1.63	Antagonism
**M2** C1 (0.65) C2 (0.14) C3 (0.21)	**0.48** **(0.35–0.55)** 0.58 0.97 2.87	5.84 ± 1.55	1.89 14.3 27.7	0.64	Synergism	**6.36** **(3.85–9.31)** 4.85 7.44 12.68	0.31 ± 0.11	1.02 7.43 7.04	1.10	Additive
**M3** C1 (0.67) C7 (0.33)	**0.63** **(0.45–0.81)** 0.58 4.73	3.36 ± 1.02	1.31 21.9	0.81	Moderate synergism	**9.01** **(6.00–12.74)** 4.85 18.99	0.22 ± 0.71	0.78 6.28	1.44	Moderate antagonism
**M5** C1 (0.74) C16 (0.26)	**0.65** **(0.52–0.70)** 0.58 88.69	3.74 ± 0.91	1.17 511.1	0.86	Slight synergism	**9.33** **(6.39–13.58)** 4.85 -	0.23 ± 0.07	0.68 123.4	1.47 *	Antagonism
**M12** C18 (0.46) C21 (0.31) C15 (0.23)	**39.22** **(30.85–49.13)** 68.65 107.95 80.35	0.06 ± 0.01	3.71 8.40 8.40	0.51	Synergism	-	**-**	-	-	-
**M20** C23 (0.74) C15 (0.26)	**2.06** **(1.18–2.92)** 2.37 80.35	0.64 ± 0.17	4.21 49.8	0.26	Strong synergism	**15.61** **(10.83–23.00)** 11.14 -	0.14 ± 0.05	0.92 27.3	1.09 *	Additive
**M21** C1 (0.50) C2 (0.50)	**0.63** **(0.45–0.83)** 0.58 0.97	2.43 ± 0.65	1.77 2.90	0.91	Additive	**8.17** **(4.88–11.78)** 4.85 7.44	0.23 ± 0.72	1.15 1.80	1.42	Moderate antagonism
**MC1** C1 (0.79) C25 (0.21)	**1.56** **(0.86–2.40)** 0.58 -	0.66 ± 0.18	0.45 732.7	2.19 *	Antagonism	**7.25** **(4.11–10.80)** 4.85 8.71	0.26 ± 0.09	0.83 5.54	1.40	Moderate antagonism
**MC6** **C26 (0.70)** C33 (0.30)	- -	**-**	-	-	-	**18.33** **(10.79–23.95)** 20.90 36.94	0.14 ± 0.05	1.59 6.62	0.78	Moderate synergism
**MC8** C29 (0.60) C11 (0.40)	**48.87** **(36.01–67.79)** - 30.46	0.06 ± 0.01	8.00 1.44	0.82 *	Slight synergism	**42.43** **(22.16–60.30)** 26.25 45.59	0.045 ± 0.02	0.97 2.54	1.42	Moderate antagonism
**C+** Dichlorvos (fumigant) Cypermethrin (contact)	2.17 (1.53–3.81)	0.51 ± 0.15				10.49 (0.10–19.96)	0.045 ± 0.02			

^a^ Concentration that caused 50% of the mortality, ^b^ slope of the linear regression of concentration–mortality, ^c^ dose reduction index ^d^ combination index, ^e^ doses that caused 50% of the mortality, and * estimated value with an LC50 = 150 mg/L for components that do not show fumigant toxicity, or with LD50 = 50 µg/adult, for components that do not show contact toxicity. Bold values indicate results obtained for mixtures.

**Table 4:** Effect of the mixtures and their components on the inhibition of AChE of *S. zeamais*.

Volatile Compounds or Mixtures [A ((Ratio):B ((Ratio):C ((Ratio)]	AChE Effect		
	${IC_{50}}^{a}\pm SD$ (mg/L)	${k_{i}}^{b}\pm SD$	Inhibitor Type
C2 (0.50)	36.60 ± 3.29	50.27 ± 0.74	C
C3 (1.00)	66.27 ± 4.35	56.76 ± 0.18	C
C7 (1.00)	18.15 ± 0.64	282.43 ± 4.14	C
C11 (0.40)	27.41 ± 1.64	159.17 ± 6.34	C
C15 (0.23)	0.19 ± 0.06	2.85 ± 0.05	NC
C16 (0.26)	26.69 ± 2.90	79.64 ± 0.55	NC
C29 (0.60)	4.24 ± 0.36	43.65 ± 3.46	C
C33 (0.30)	7.72 ± 0.78	198.55 ± 3.51	C
M5 [C1 (0.74):C16 (0.26)]	30.19 ± 5.78	207.85 ± 2.05	C
M12 [C18 (0.46):C21 (0.31):C15 (0.23)]	0.81 ± 0.06	3.27 ± 0.06	NC
M20 [C23 (0.74):C15 (0.26)]	0.61 ± 0.05	1.44 ± 2.98 × 10^−3^	C
M21 [C1 (0.50):C2 (0.50)]	30.24 ± 4.60	159.15 ± 7.35	C
MC1 [C1 (0.79):C25 (0.21)]	22.32 ± 6.78	94.46 ± 3.57	C
MC6 [C26 (0.70):C33 (0.30)]	4.45 ± 0.68	30.45 ± 3.51	C
MC8 [C29 (0.60):C11 (0.40)]	5.28 ± 0.72	44.61 ± 1.64	C
C+	9.60 × 10^−3^ ± 2.08 × 10^−3^	**-**	-

Inhibitor type. C: competitive inhibitor, NC: non-competitive inhibitor. ^a^ Concentration that caused 50% of inhibition, and ^b^ the inhibitory constant (Ki) represents the inhibitor concentration required to achieve half of the maximum inhibition.

**Table 5:** Insecticidal effect and interaction of chemical constituents of EOs against *S. zeamais*, based on the median effect of the law of mass action.

Mixes A (Ratio) B (Ratio) C (Ratio)	Interaction of Components in Mixtures		
	DRI ^a^	CI ^b^	Interaction
M5		0.21	Strong synergism
C1 (0.74)	109.80		
C16 (0.26)	5.10		
M12		1.95	Antagonism
C18 (0.46)	4 268.45		
C21 (0.31)	786.24		
C15 (0.23)	0.51		
M20		2.31	Antagonism
C23 (0.74)	42 279.60		
C15 (0.26)	0.43		
M21		0.29	Strong synergism
C1 (0.50)	162.21		
C2 (0.50)	3.53		
MC1		0.04	Very strong
C1 (0.79)	139.10		synergism
C25 (0.21)	28.14		
MC6		0.13	Strong synergism
C26 (0.70)	81.17		
C33 (0.30)	8.75		
MC8		0.52	Synergism
C29 (0.60)	2.37		
C11 (0.40)	9.70		

^a^ Dose reduction index, ^b^ combination index.

## Data Availability

The original contributions presented in this study are included in the article/Supplementary Material. Further inquiries can be directed to the corresponding authors.

## Supplementary Materials

The following are available online at https://www.mdpi.com/article/10.3390/insects16060609/s1, Table S1. Sample information of the 51 EO-derived volatile compounds (VCs) worked.; Table S2. Physical and spectroscopic characterization of estragole (43) isolated from *Artemisia dracunculus* [86]; Table S3. Physical and spectroscopic characterization of piperitone (27) isolated from *Piper aduncum* [87]; Table S4. Synthesis of isopulegone (28) from Isopulegol, physical and spectroscopic characterization [88]; Table S5. Characteristics of the volatile compounds for cluster analysis of fumigant and contact toxicity; Table S6. Criteria for the predesign of ternary mixtures with fumigant toxicity on *S. zeamais;* Table S7. Criteria for the predesign of ternary mixtures with contact toxicity on *S. zeamais;* Table S8. Analysis of variance of RSM, including diagnostic statistics for the affected fraction of *S. zeamais* for matrices with three compounds with fumigant effect; Table S9. Analysis of variance of RSM, including diagnostic statistics for the affected fraction of *S. zeamais* for matrices with three compounds with contact toxic effect; Table S10. characterization of protein extract from *S. zeamais*; Figure S1. ^1^H NMR Spectra (400 MHz, CDCl_3_) of estragole; Figure S2. APT Spectra (100 MHz, CDCl_3_) of estragole; Figure S3. ^1^H NMR Spectra (400 MHz, CDCl_3_) of piperitone; Figure S4. APT Spectra (100 MHz, CDCl_3_) of piperitone; Figure S5. ^1^H NMR Spectra (400 MHz, CDCl_3_) of isopulegone; Figure S6. APT Spectra (100 MHz, CDCl_3_) of isopulegone; Figure S7. Graphs of the fumigant toxicity interaction of the components of the mixtures using the median effect model of the law of mass action; Figure S8. Graphs of the contact toxicity interaction of the components of the mixtures using the median effect model of the law of mass action; Figure S9. Dose-effect curves of *S. zeamais* AChE inhibition of the mixtures and their components; Figure S10. Lineweaver-Burk plots of the kinetic study of mixtures and their components on AChE on *S. zeamais*; Figure S11. Graphs of the interaction of the components of the mixtures using the median effect model of the law of mass action.
