# Supplementary material for: Synergistic Insecticidal Activity of Plant Volatile Compounds: Impact on Neurotransmission and Detoxification Enzymes in Sitophilus zeamais
Source: Insects. 2025 Jun 9;16(6):609. doi: 10.3390/insects16060609 (PMC12194779; doi:10.3390/insects16060609)
Supplement: Supplementary file 1 [file insects-16-00609-s001.zip › insects-3558905-supplementary.pdf]

insects

MDPI

Supplementary material

Synergistic Insecticidal Activity of Plant Volatile Compounds: Impact on Neurotransmission and Detoxification Enzymes in *Sitophilus zeamais*

Leidy J. Nagles Galeano[1], Juliet A. Prieto-Rodríguez[2*] and Oscar J. Patiño-Ladino[1]

Table S1. Sample information of the 51 EO-derived volatile compounds (VCs) worked.

N°	Compound	Selection Criteria	Purity (%)	Acquisition
**1**	β-Pinene	1	98.0	Merck ®, Bogotá, Colombia
**2**	α-Terpinene	2	90.0	Sigma Aldrich ® Saint Louis, MO, USA
**3**	DL-Limonene (1:1)	1	95.0	Merck ®, Bogotá, Colombia
**4**	p-Cymene	1	90.0	Merck ®, Bogotá, Colombia
**5**	R-(-)-Terpinen-4-ol	3*	95.0	Sigma Aldrich ® Saint Louis, MO, USA
**6**	1R,2S,5R-Isopulegol	4	98.0	Sigma Aldrich ® Saint Louis, MO, USA
**7**	1,8-Cineole	1,2	99.0	Sigma Aldrich ® Saint Louis, MO, USA
**9**	1R-(-)-Fenchone	1	98.0	Sigma Aldrich ® Saint Louis, MO, USA
**9**	R-(+)-Pulegone	1	90.0	Sigma Aldrich ® Saint Louis, MO, USA
**10**	S-(-)-Pulegone	1	98.0	Sigma Aldrich ® Saint Louis, MO, USA
**11**	S-(+)-Carvone	1	96.0	Sigma Aldrich ® Saint Louis, MO, USA
**12**	R-(-)-Carvone	1	98.0	Sigma Aldrich ® Saint Louis, MO, USA
13	Piperitone	1	96.0	Isolated from *Piper aduncum.*
14	2S,5R-Iisopulegone	3	97.0	Synthesis from 1R,2S,5R-Isopulegol
15	αβ-Thujone (70:10)	4	79.9	Sigma Aldrich ® Saint Louis, MO, USA
16	Estragole	1	95.0	Isolation from *Artemisia dracunculus*
17	2-Nonanone	4	99.0	Sigma Aldrich ® Saint Louis, MO, USA
**18**	α-Pinene	1	97.0	Merck ®, Bogotá, Colombia
**19**	δ-3-Carene	1	90.0	Sigma Aldrich ® Saint Louis, MO, USA
**20**	R-(-)-α-Phellandrene	1	95.0	Sigma Aldrich ® Saint Louis, MO, USA
**21**	Terpinolene	1	85.0	Sigma Aldrich ® Saint Louis, MO, USA
22	n-Nonane	3	99.0	Merck ®, Bogotá, Colombia
**23**	Sabinene	1	75.0	Sigma Aldrich ® Saint Louis, MO, USA
**24**	γ-Terpinene	1	97.0	Sigma Aldrich ® Saint Louis, MO, USA
**25**	Alcanphor	1,2	96.0	Sigma Aldrich ® Saint Louis, MO, USA
**26**	Citronellal	1	93.5	Merck ®, Bogotá, Colombia
27	2-Undecanone	2	98.0	Sigma Aldrich ® Saint Louis, MO, USA
28	4-Undecanone	4	98.0	Sigma Aldrich ® Saint Louis, MO, USA
**29**	Thymol	1	99.5	Sigma Aldrich ® Saint Louis, MO, USA
**30**	Carvacrol	1	98.0	Sigma Aldrich ® Saint Louis, MO, USA
**31**	α-Terpineol	1	96.0	Alpha Aesar ®, England, UK
32	Geranyl acetate	1	97.0	Sigma Aldrich ® Saint Louis, MO, USA
**33**	Linalool	1	97.0	Merck ®, Bogotá, Colombia
34	Terpinyl acetate	1	96.0	Sigma Aldrich ® Saint Louis, MO, USA
35	Safrole	2	97.0	Sigma Aldrich ® Saint Louis, MO, USA
36	L-Menthyl acetate	3	98.0	Sigma Aldrich ® Saint Louis, MO, USA
37	Farnesene	2	97.0	Sigma Aldrich ® Saint Louis, MO, USA
38	Isoeugenol	4	98.0	Merck ®, Bogotá, Colombia
39	trans-Anethole	1	98.0	Merck ®, Bogotá, Colombia
40	2-Decanone	4	98.0	Sigma Aldrich ® Saint Louis, MO, USA
**41**	Geraniol	4	97.0	Alpha Aesar ®, England, UK
**42**	Citral	1	95.0	Sigma Aldrich ® Saint Louis, MO, USA
43	Linalyl acetate	4	98.0	Sigma Aldrich ® Saint Louis, MO, USA
44	β-Caryophyllene	1,2	80.0	Sigma Aldrich ® Saint Louis, MO, USA
45	Nerolidol	4	98.0	Merck ®, Bogotá, Colombia
45	Farnesol	2	97.0	Merck ®, Bogotá, Colombia
47	Eugenol	2	99.0	Sigma Aldrich ® Saint Louis, MO, USA
48	Methyl eugenol	2	90.0	Merck ®, Bogotá, Colombia
49	Methyl isoeugenol	3	98.0	Sigma Aldrich ® Saint Louis, MO, USA
50	Decanal	3	96.0	Sigma Aldrich ® Saint Louis, MO, USA
51	Nonanal	1	95.0	Sigma Aldrich ® Saint Louis, MO, USA

Selection criteria: 1. Majority components (≥ 4.0%), 2. Minority components present in two or more bioactive EOs, 3. Minority components without bibliographic reports, 4. Components were structurally similar to the selected compounds.

**Compounds with reported inhibitory effects on one of the insect's enzymes of interest.*

Table S2 Physical and Spectroscopic Characterization of Estragole Isolated from Artemisia dracunculus. (43)

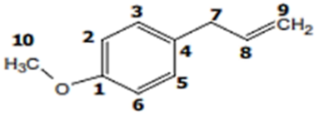	**Name**	Estragole
	**Physical State**	Liquid
	**Molecular Formula**	$C_{10}H_{12}O$
	**Molecular Mass:**	148.2 g/mol
**^{1}H NMR (400 MHz, $CDCl_3$) δ (ppm)** 7.11 (d, *J* = 8.7 Hz, 2H, H-3 y H-5), 6.86 (d, *J* = 8.6 Hz, 2H, H-2 y H-6), 5.96 – 6.06 (m, 1H, H-8), 5.08 - 5,14 (m, 2H, H-9), 3.81 (s, 3H, OCH3) y 3.34 (d, J = 6.7 Hz, 2H, H-7).		
**APT (101 MHz, $CDCl_3$) δ (ppm)** 113.8 (CH-2, CH-6), 129.5 (CH-3, CH-5), 132.1 (C-4), 158.0 (C-1), 39.4 (CH_2-7), 115.4 (CH_2-9) y 137.9 (CH_2-8).		
**Data described in the literature** [86]		

Figure S1 ^{1}H NMR Spectra (400 MHz, $CDCl_3$) of Estragole

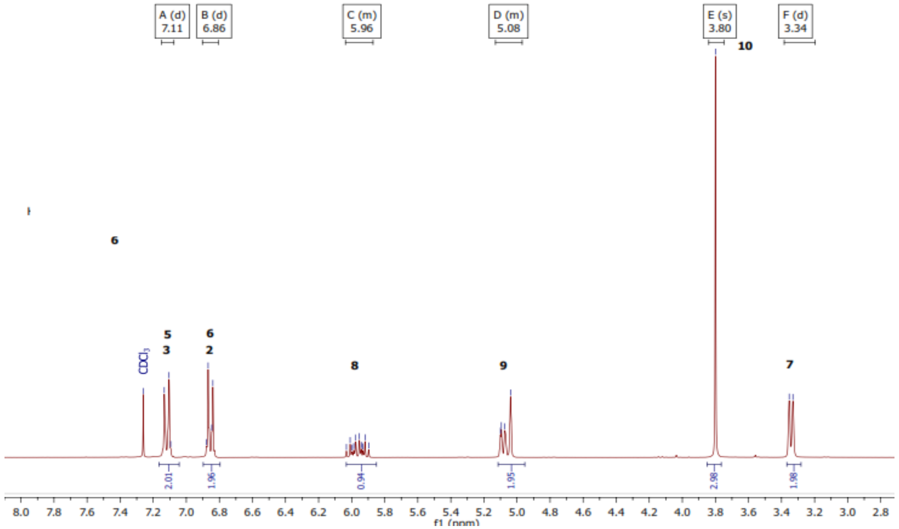

Figure S2 APT Spectra (100 MHz, $CDCl_3$) of Estragole

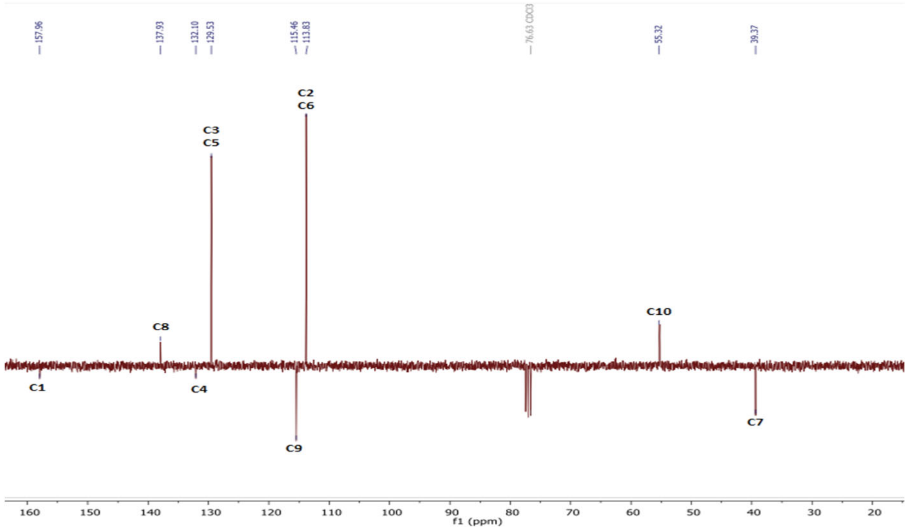

Table S3 Physical and Spectroscopic Characterization of Piperitone Isolated from Piper aduncum (27).

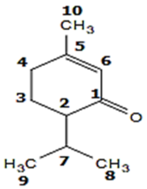	**Name**	Piperitone
	**Physical State**	Colorless Liquid
	**Molecular Formula**	$C_{10}H_{16}O$
	**Molecular Mass:**	152.23 g/mol
**^{1}H NMR (400 MHz, $CDCl_3$) δ (ppm)** 5.83 (dq, J = 2.9, 1.4 Hz, 1H, H-6), 2.40 – 2.23 (m, 3H H-2 y H-4), 2.04 – 1.93 (m, 2H, 3-H), 1.91 (dd, J = 2.1, 1.0 Hz, 3H, H-10), 1.86 – 1.72 (m, 1H, H-7), 0.93 (d, J = 7.0 Hz, 3H, H-8) y 0.84 (d, J = 6.8 Hz, 3H, H-9).		
**APT (101 MHz, $CDCl_3$) δ (ppm)** 201.5(C=O), 161.3(C-7), 126.9(C-8), 51.7 (C-6), 30.5 (C-2), 25.9(C-3),24.2 (C-4), 23,0(C-5), 20,8 (C-9) y 18.7 (C-10).		

**Data described in the literature [87]**

Figure S3 ^1H NMR Spectra (400 MHz, $CDCl_3$) of Piperitone

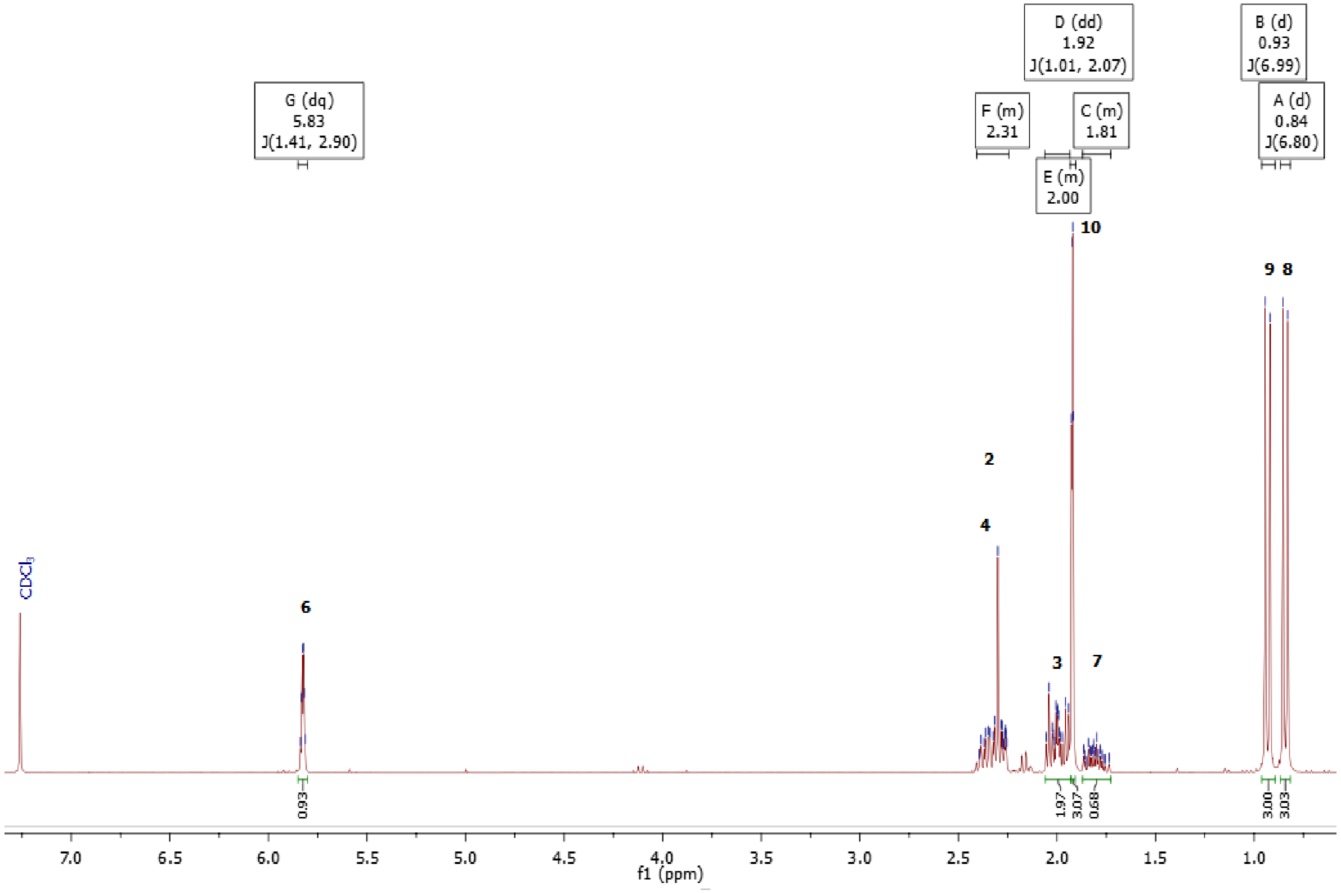

Figure S4 APT Spectra (100 MHz, $CDCl_3$) of Piperitone

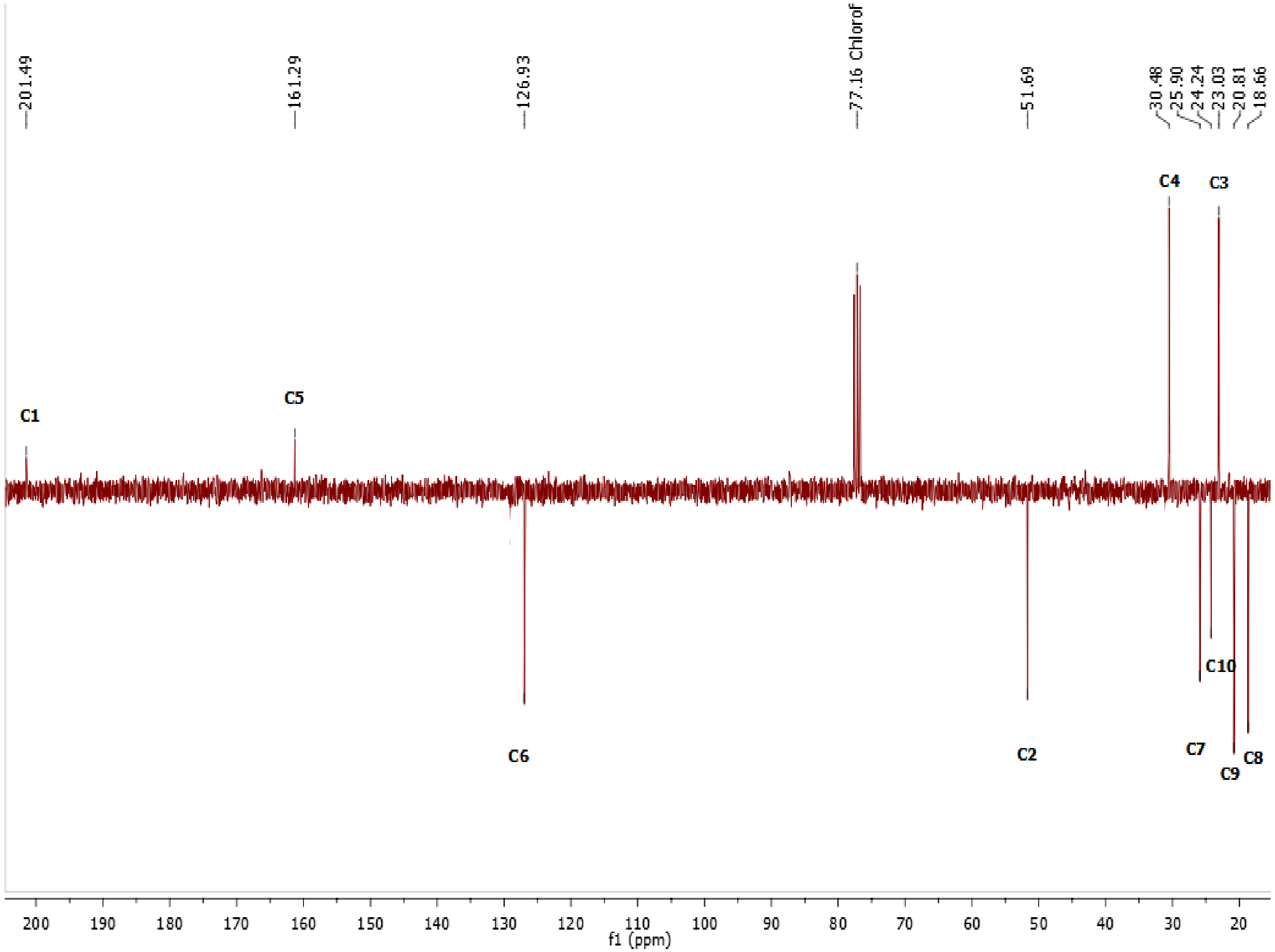

Table S4 Synthesis of Isopulegone (28) from Isopulegol, Physical and Spectroscopic Characterization

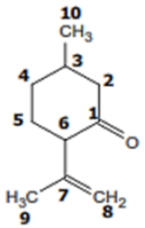	**Name**	2S,5R-Iisopulegone
	**Physical State**	Colorless Liquid
	**Molecular Formula**	$C_{10}H_{16}O$
	**Molecular Mass:**	152,23 g/mol
**^{1}H NMR (400 MHz, $CDCl_3$) δ (ppm)** 4.92 (s, 1H, H-8), 4.70 (s, 1H, H-8), 2.94 (dd, J 13.1, 5.4 Hz, 1H, H-6), 2.40 (ddd, J =13.3, 3.6, 2.4, 1H, H_{ec}-2), 2.09 – 1.97 (m, 2H, H_{ax}-2 y H_{ec}-5), 1.96 – 1.83 (m, 2H, H_{ec}-4 y H-3), 1.80 (qd, J 13.1, 3.3, 1H, H_{ax}-5), 1.73 (s, 3H, CH_3-9), 1.49 – 1.30 (m, 1H, H_{ax}-4) y 1.02 (d, J 6.24, 3H, CH_3-10).		
**APT (101 MHz, $CDCl_3$) δ (ppm)** (101 MHz, CDCl3) δ = 210.3 (C=O), 143.6 (C-7), 112.9 (C-8), 57.8 (C-6), 50.7 (C-2), 35.4 (C-3), 34.0 (C-4), 31.3 (C-5), 22.4 (C-9) y 21.4 (C-10).		
**Synthesis:** For the synthesis of **Isopulegone (28)**, (1R,2S,5R)-isopulegol (1.00 g, 6.54 mmol) was added to a suspension of pyridinium chlorochromate (PCC) (2.10 g, 9.72 mmol) in 30 mL of dry dichloromethane. The resulting mixture was stirred for 18 hours at 25 °C under a nitrogen atmosphere. After this period, the reaction mixture was filtered through Celite, and the organic phase was removed by reduced-pressure distillation. The resulting crude product (1.12 g) was purified by column chromatography using CH_2Cl_2 as the elution system, yielding a colorless oil corresponding to **isopulegone S-1** (880.0 mg, 89.2%). The product was identified by NMR spectral analysis and comparison with literature data [**88**].		
**Data described in the literature** [**88**]		

Figure S5 ^{1}H NMR Spectra (400 MHz, $CDCl_3$) of Isopulegone

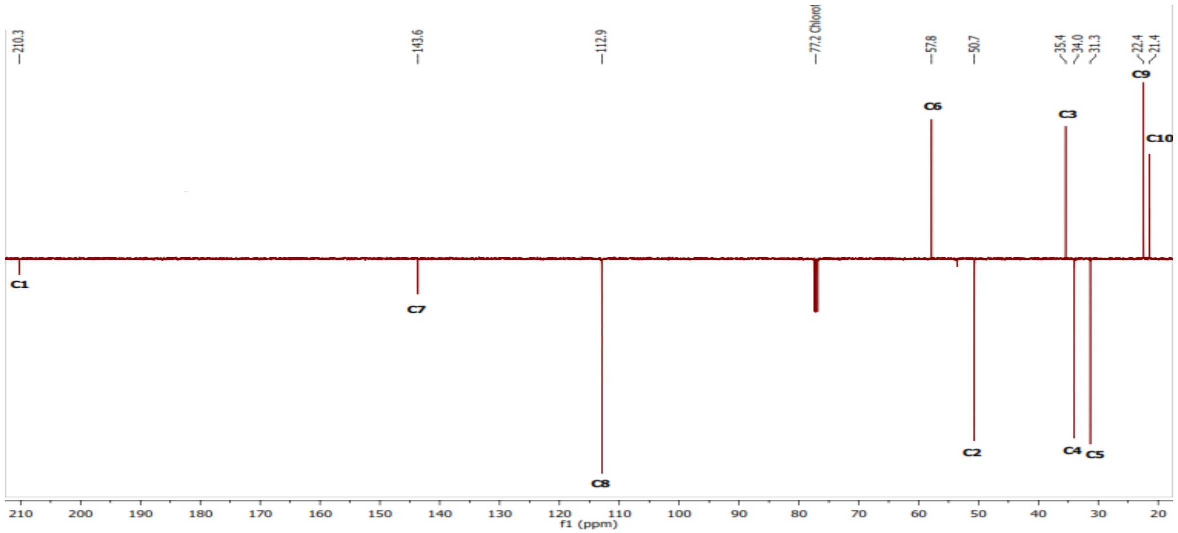

Figure S6 APT Spectra (100 MHz, $CDCl_3$) of Isopulegone

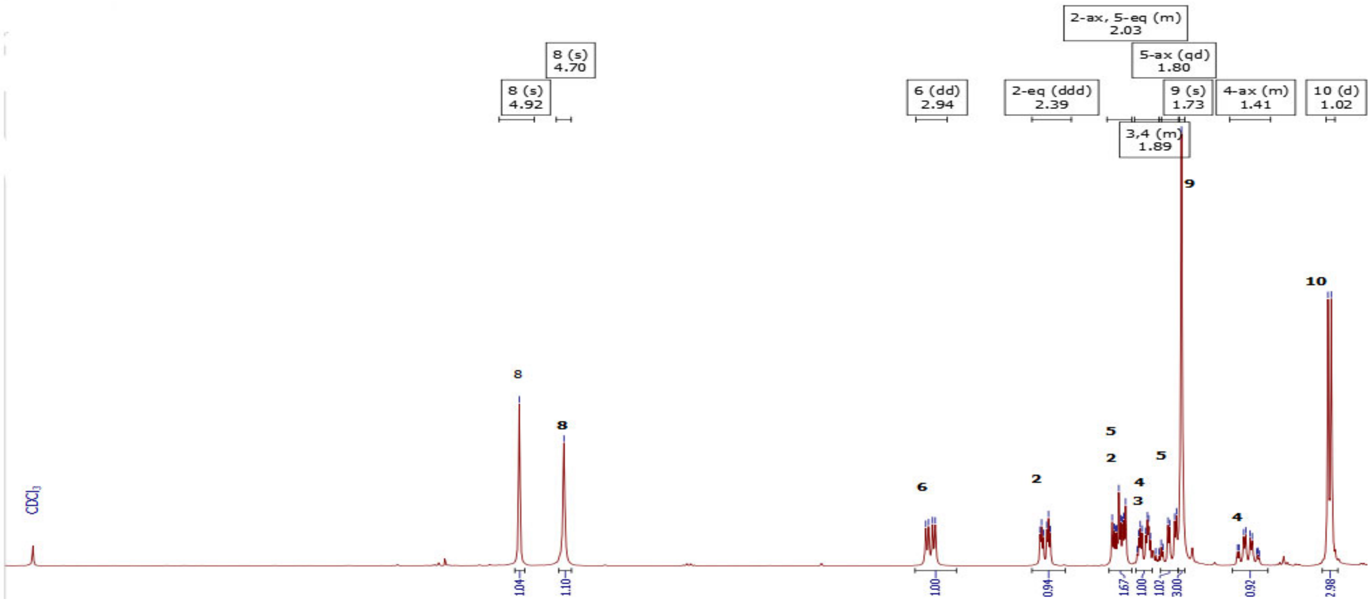
2-ax, 5-eq (m)
2.03
8 (s)
4.70
5-ax (qd)
1.80
8 (s)
4.92
6 (dd)
2.94
2-eq (ddd)
2.39
9 (s)
1.73
4-ax (m)
1.41
10 (d)
1.02
3,4 (m)
1.89
CDCl3
1.04
1.10
1.00
0.94
1.67
1.00
1.02
3.00
0.92
2.98

Table S5. Characteristics of the volatile compounds for cluster analysis of fumigant and contact toxicity

Compound	C	Parameters for fumigant cluster					Parameters for contact cluster				Traits to characterize clusters				
		LC_{30}	LC_{50}	LC_{90}	slope	carbon skeleton	LD_{30}	LD_{50}	LD_{90}	slope	Compound Type	biosynthetic precursor	organic Function	Vapour pressure (Pa)	partition coefficient ($LogK_{ow}$)
**S-(-)-Pulegone**	C1	0.44	0.58	0.93	3.72	Monocyclic	3.81	4.85	7.40	0.50	Monoterpenoid	α-Terpinyl cation	Ketone	16.4	3.08
**S-(+)-Carvone**	C2	0.68	0.97	1.68	1.80	Monocyclic	6.54	7.44	9.64	0.58	Monoterpenoid	α-Terpinyl cation	Ketone	16.4	3.08
**R-(-)-Carvone**	C4	1.12	1.42	2.16	1.73	Monocyclic	12.16	16.89	28.44	0.11	Monoterpenoid	α-Terpinyl cation	Ketone	13.7	2.71
**2S,5R-Iisopulegone**	C23	1.76	2.37	3.88	0.85	Monocyclic	8.19	10.99	17.83	0.19	Monoterpenoid	α-Terpinyl cation	Ketone	18.7	2.85
**R-(-)-Terpinen-4-ol**	C5	1.04	2.82	7.15	0.30	Monocyclic	17.50	19.66	24.94	0.24	Monoterpenoid	Terpinen-4-yl cation	Alcohol	6.2	3.26
**S-(+)-Carvone**	C3	1.87	2.87	5.32	0.52	Monocyclic	8.36	12.68	23.24	0.12	Monoterpenoid	α-Terpinyl cation	Ketone	13.3	2.71
**αβ-Thujone**	C6	3.64	4.38	6.19	0.52	Bicyclic	26.88	32.04	44.67	0.10	Monoterpenoid	Terpinen-4-yl cation	Ketone	54.9	2.52
**Piperitone**	C22	1.04	4.73	7.15	0.71	Monocyclic	7.31	9.45	14.70	0.24	Monoterpenoid	α-Terpinyl cation	Ketone	15.9	2.85
**1R,2S,5R-Isopulegol**	C7	7.19	9.09	13.73	0.52	Monocyclic	17.50	18.99	24.94	0.17	Monoterpenoid	α-Terpinyl cation	Alcohol	9.3	2.36
**2-Nonanone**	C9	3.33	4.34	6.81	0.28	Acyclic	28.65	31.85	39.69	0.16	Aliphatic	Nonaketid	Ketone	83.2	3.14
**1R-(-)-Fenchone**	C8	7.95	10.59	17.06	0.20	Bicyclic	29.05	34.65	48.33	0.09	Monoterpenoid	Pinyl cation	Ketone	108.9	3.52
**1.8-Cineole**	C10	9.4	12.97	21.69	0.15	Bicyclic	47.51	56.82	79.58	0.06	Monoterpenoid	α-Terpinyl cation	Ether	253.3	2.74
**p-Cymene**	C12	21.2	28.68	46.96	0.07	Aromatic					Monoterpenoid	α-Terpinyl cation	Hydrocarbon	96.3	
**Estragole**	C11	19.42	30.47	57.46	0.05	Aromatic	31.11	45.07	79.17	0.04	Phenylpropanoid	Coumaryl acetate	Ether	22.7	3.47
**Terpinoleno**	C24	45.53	60.24	96.19	0,03	Monocyclic					Monoterpenoid	Terpinen-4-yl cation	Hydrocarbon	98.7	
**α-Terpinene**	C13	37.11	52.12	88.83	0.04	Monocyclic					Monoterpenoid	Terpinen-4-yl cation	Hydrocarbon	145.3	
**Sabinene**	C18	50.93	66.43	104.3	0.03	Bicyclic					Monoterpenoid	Terpinen-4-yl cation	Hydrocarbon	351.0	
**δ-3-Carene**	C15	57.81	80.44	135.75	0.02	Bicyclic					Monoterpenoid	α-Terpinyl cation	Hydrocarbon	464.0	
**DL-limonene**	C16	69.28	88.69	136.14	0.03	Monocyclic					Monoterpenoid	α-Terpinyl cation	Hydrocarbon	206.6	
**R-(-)-α-Phellandrene**	C14	66.91	88.87	142.53	0.02	Monocyclic					Monoterpenoid	α-Terpinyl cation	Hydrocarbon	186.7	
**β-Pinene**	C17	75.6	97.6	151.34	0.02	Bicyclic					Monoterpenoid	Pinyl cation	Hydrocarbon	390.6	

**γ- Terpinene**	C21	75.92	106.97	182.86	0.02	Monocyclic					Monoterpenoid	α-Terpinyl cation	Hydrocarbon	145.3	
**N-nonane**	C20	83.7	108.98	170.75	0.02	Acyclic					Monoterpenoid	Nonaketid	Alcano	593.3	
**α-Pinene**	C19	82.38	110.38	178.81	0.02	Bicyclic					Monoterpenoid	Pinyl cation	Hydrocarbon	633.3	
**Carvacrol**	C25					Aromatic	6.76	8.71	13.45	0.27	Monoterpenoid	α-Terpinyl cation	Alcohol		3.49
**Eugenol**	C26					Aromatic	16.85	20.90	30.82	0.13	Phenylpropanoid	Coniferyl acetate	Ether/Alcohol		2.49
**Citral**	C27					Acyclic	16.83	21.43	32.68	0.11	Monoterpenoid	Geranyl cation	Aldehyde		2.76
**Linalool**	C28					Acyclic	16.00	21.87	36.23	0.09	Monoterpenoid	Neryl cation	Alcohol		2.97
**trans-anetol**	C29					Aromatic	18.13	26.25	46.08	0.07	Monoterpenoid	Coumaryl acetate	Ether		3.13
**Geraniol**	C30					Acyclic	22.83	28.14	41.13	0.10	Monoterpenoid	Geranyl cation	Alcohol		3.56
**Isoeugenol**	C31					Aromatic	13.92	29.10	66.20	0.04	Phenylpropanoid	Coniferyl acetate	Ether/Alcohol		3.04
**Safrole**	C32					Aromatic	17.19	36.31	83.04	0.03	Phenylpropanoid	Coniferyl acetate	Ether		3.02
**Citronellal**	C33					Acyclic	31.33	36.94	50.64	0.09	Monoterpenoid	Geranyl cation	Aldehyde		3.58
**4-undecanone**	C34					Acyclic	30.13	37.04	53.95	0.08	Aliphatic	Nonaketid	Ketone		4.05
**2-decanone**	C35					Acyclic	30.20	38.03	57.17	0.07	Aliphatic	Nonaketid	Ketone		3.73
**2-undecanone**	C36					Acyclic	37.01	42.86	57.13	0.09	Aliphatic	Nonaketid	Ketone		4.09
**Geranyl acetate**	C37					Acyclic	51.28	73.00	126.10	0.02	Monoterpenoid	Geranyl cation	Ester		4.04
**Dichlorvos**	C+	1.05	2.17	4.57	0.51	Acyclic					-	-	-	1.60	
**Cypermethrin**	C+					Bicyclic	4.16	8.55	19.27	0.12	-	-	-		

Table S6. Criteria for the predesign of ternary mixtures with fumigant toxicity on *S. zeamais*

Mix Code	ternary mixtures (A + B + C)			Criteria for the predesing of mixtures (compounds belonging to the same group and between different groups, structural characteristics of the members of each group, and reported chemical composition of the bioactive EOs)
M1	C1	C3	C4	Monoterpene ketones from cluster G1, derived from the α-terpinyl cation, differing in the position of the main organic function and stereochemistry (carvone).
M2	C1	C2	C3	Monoterpene ketones from cluster G1, differing in the position of the main organic function.
M3	C1	C3	C7	Monocyclic monoterpenoids from cluster G1, derived from the α-terpinyl cation, differing in substituent (ketone/alcohol).
M4	C1	C3	C5	Monocyclic monoterpenoids from cluster G1, differing in biosynthetic precursor and substituent (ketone/alcohol).
M5	C1	C3	C16	Monocyclic monoterpenoids from clusters G1 and G3, constituents of L. alba (C3-C16) and M. septentrionalis (C1, C16) EOs, differing in substituent (no substituents/ketone).
M6	C22	C3	C16	Monocyclic monoterpenoids from clusters G1 and G3, constituents of L. alba EOs, differing in position and type of substituent (no substituents/ketone).
M7	C8	C3	C10	Monoterpenoids from cluster G1, differing in carbon skeleton, biosynthetic precursor, and substituent (ketone/ether).
M8	C8	C7	C6	Monoterpenoids from cluster G1, differing in carbon skeleton, biosynthetic precursor, and substituent (ketone/alcohol).
M9	C12	C9	C10	Compounds from clusters G1 and G2, constituents of Xylopia discreta EOs (C12, C10).
M10	C20	C19	C17	Monoterpenoids from clusters G1 and G3, constituents of Eucalyptus sp. and Ocotea sp. EOs (C19, C17).
M11	C10	C16	C19	Monoterpenoids from clusters G1 and G3, constituents of Eucalyptus sp. and Ocotea sp. EOs (C19, C16).
M12	C18	C21	C15	Monoterpenoids with hydrocarbon function, from clusters G2 and G3, constituents of C. sempervirens and Z. monophyllum EOs (C18, C21).
M13	C14	C12	C16	Monoterpenoids with hydrocarbon function, from clusters G2 and G3, constituents of P. el-metanum and C. album EOs (C12, C14).
M14	C10	C16	C19	Monoterpenoids from clusters G1 and G3, constituents of Eucalyptus sp. and Ocotea sp. EOs (C19, C16).
M15	C11	C12	C13	Compounds from clusters G2 and G3, constituents of C. sempervirens EO (C12, C13).
M16	C10	C16	C18	Monoterpenoids from clusters G1, G2, and G3, constituents of Xylopia discreta EO, based on M12 results, differing in substituent (no substituent/ether).
M17	C10	C5	C18	Monoterpenoids from clusters G1, G2, and G3, constituents of Z. monophyllum EO (C10, C18), based on M12 results, differing in substituent (no substituent/alcohol/ether).
M18	C1	C22	C18	Monoterpenoids from clusters G1 and G2, constituents of M. septentrionalis EO (C1, C18), based on M12 results, differing in substituent (no substituent/ketone).
M19	C3	C15	C18	Monoterpenoids from clusters G1 and G2, constituents of C. sempervirens EO (C15, C18), based on M12 results, differing in substituent (no substituent/ketone).
M20	C6	C23	C15	Monoterpenoids from clusters G1 and G2, based on M12 results, differing in substituent (substituent/ketone).

Table S7 Criteria for the predesign of ternary mixtures with contact toxicity on *S. zeamais*

Mix Code	ternary mixtures (A + B + C)			Criteria for the predesing of mixtures (compounds belonging to the same group and between different groups, structural characteristics of the members of each group, and reported chemical composition of the bioactive EOs)
**MC1**	**C1**	**C25**	**C26**	Compounds from clusters G1 and G2, constituents of Lippia sp. EO (C25, C26), differing mainly in carbon skeleton (monocyclic/aromatic).
**MC2**	**C9**	**C25**	**C26**	Compounds from clusters G1, G2, and G3, constituents of Lippia sp. EO (C24, C25), differing mainly in carbon skeleton (acyclic/aromatic).
**MC3**	**C1**	**C25**	**C34**	Compounds from clusters G1 and G3, constituents of Lippia sp. EO (C25, C26), differing mainly in carbon skeleton (monocyclic/aromatic).
**MC4**	**C25**	**C26**	**C34**	Compounds from clusters G1 and G3, constituents of Lippia sp. EO (C25, C26), differing mainly in carbon skeleton (acyclic/aromatic).
**MC5**	**C1**	**C5**	**C10**	Monoterpenoids from clusters G1 and G3, constituents of Z. monophyllum (C5, C10) and S. viminea (C1, C10) EOs, differing in biosynthetic precursor.
**MC6**	**C25**	**C37**	**C33**	Monoterpenoids from clusters G2 and G3, constituents of C. nardus EO.
**MC7**	**C27**	**C37**	**C28**	Monoterpenoids from clusters G1 and G3, constituents of C. citratus EO.
**MC8**	**C29**	**C11**	**C28**	Compounds from clusters G2 and G3, constituents of I. verum (C29, C11) and O. basilicum (C11, C28) EOs.
**MC9**	**C22**	**C10**	**C28**	Monoterpenoids from clusters G1 and G3, constituents of P. asperiusculum (C22, C10) and P. aduncum (C22, C28) EOs.

Table S8. Analysis of variance of RSM, including diagnostic statistics for the affected fraction of *S. zeamais* for matrices with three compounds with fumigant effect

Table S8-1 ANOVA RSM of mix M1

Source	gl	SC	CM	F	ϱ	Coef
Model	5	290.33	56.64	271.88	0.000	
Linear	2	231.63	54.25	260.40	0.000	
Quadratic	3	58.70	18.49	88.76	0.000	
C1 * C3	1	16.51	12.04	57.80	0.000	9.50
C1 * C4	1	22.01	15.04	72.20	0.000	9.50
C3 * C4	1	20.16	20.16	96.80	0.000	-11.00
R. Error	18	3.75	0.20			
Cor Total	23	293.83				

Table S8-2 ANOVA RSM of mix M2

Source	gl	SC	CM	F	ϱ	Coef
Model	5	66.33	13.27	68.23	0.000	
Linear	2	44.43	24.25	124.71	0.000	
Quadratic	3	21.90	7.30	37.54	0.000	
C1 * C2	1	0.00	1.04	5.36	0.030	2.50
C1 * C3	1	8.400	12.04	61.93	0.000	8.50
C2 * C3	1	13.50	13.50	69.43	0.000	8.33
R. Error	18	3.50	0.194			
Cor Total	23	69.83				

R^2	0.987
R^2_{pred}	0.977
R^2_{aju}	0.986

R^2	0.950
R^2_{pred}	0.911
R^2_{aju}	0.936

Table S8-3 ANOVA RSM of mix M3

Source	gl	SC	CM	F	ϱ	Coef
**Model**	5	329.19	65.84	25.75	0.000	
**Linear**	2	247.91	60.59	23.70	0.000	
**Quadratic**	3	81.27	27.09	10.60	0.000	
**C1 * C3**	1	15.62	18.94	7.41	0.012	10.65
**C1 * C7**	1	58.20	46.61	18.23	0.000	15.99
**C3 * C7**	1	7.44	7.44	2.91	0.102	-6.60
**R. Error**	17	56.24	2.56			
**Cor Total**	27	385.43				
**R^2**		0.854				
**R^2_{pred}**		0.795				
**R^2_{aju}**		0.821				

Table S8-4 ANOVA RSM of mix M5

Source	gl	SC	CM	F	ϱ	Coef
Model	5	381.33	76.27	305.07	0.000	
Linear	2	288.03	76.75	307.00	0.000	
Quadratic	3	93.30	31.10	124.40	0.000	
C18 * C21	1	8.23	12.04	48.17	0.000	8.50
C18 * C15	1	74.40	63.37	253.50	0.000	19.50
C21 * C15	1	10.67	10.67	42.67	0.000	-8.00
R. Error	18	4.50	0.25			
Cor Total	23	385.83				
R^2		0.988				
R^2_{pred}		0.979				
R^2_{aju}		0.982				

Table S8-5 ANOVA RSM of mix M6

Source	gl	SC	CM	F	ϱ	Coef
**Model**	5	421.208	84.242	404.36	0.000	
**Linear**	2	304.933	114.083	547.60	0.000	
**Quadratic**	3	116.275	38.758	186.04	0.000	
**C1 * C3**	1	57.604	40.042	192.20	0.000	15.50
**C1 * C16**	1	1.63	0.000	0.00	1.000	$5.7x10^{-16}$
**C3 * C16**	1	57.04	57.042	273.80	0.000	-18.50
**R. Error**	18	3.75	0.208			
**Cor Total**	23	424.96				
**R^2**		0.991				
**R^2_{pred}**		0.984				
**R^2_{aju}**		0.989				

Table S8-6 ANOVA RSM of mix M7

Source	gl	SC	CM	F	ϱ	Coef
**Model**	Model	5	121.33	24.27	97.07	0.000
**Linear**	Linear	2	100.23	24.33	97.33	0.000
**Quadratic**	Quadratic	3	21.10	7.03	28.13	0.000
**C1 * C18**	C8 * C3	1	8.93	8.17	32.67	0.000
**C1 * C22**	C8 * C10	1	8.00	6.00	24.00	0.000
**C22 * C18**	C3 * C10	1	4.17	4.17	16.67	0.001
**R. Error**	R. Error	18	4.50	0.25		
**Cor Total**	Cor Total	23	125.83			
**R^2**	R^2		0.964			
**R^2_{pred}**	R^2_{pred}		0.936			
**R^2_{aju}**	R^2_{aju}		0.954			

Table S8-7 ANOVA RSM of mix M12

Source	gl	SC	CM	F	ϱ	Coef
**Model**	5	119.21	23.84	49.05	0.000	
**Linear**	2	16.23	9.33	19.20	0.000	
**Quadratic**	3	102.96	34.32	70.61	0.000	
**C22 * C3**	1	65.09	80.67	165.94	0.000	22.00
**C22 * C16**	1	35.22	37.50	77.14	0.000	15.00
**C3 * C16**	1	2.67	2.67	5.49	0.031	4.00
**R. Error**	18	8.75	0.49			
**Cor Total**	23	127.96				
**R^2**		0.932				
**R^2_{pred}**		0.878				
**R^2_{aju}**		0.913				

Table S8-8 ANOVA RSM of mix M14

Source	gl	SC	CM	F	ϱ	Coef
**Model**	5	268.71	53.74	227.61	0.000	
**Linear**	2	179.43	96.58	409.06	0.000	
**Quadratic**	3	89.27	29.76	126.04	0.000	
**C6 * C23**	1	20.09	30.37	128.65	0.000	13.50
**C6 * C15**	1	2.52	0.042	0.18	0.697	-0.50
**C23 * C15**	1	66.67	66.67	282.35	0.000	20.00
**R. Error**	18	4.25	0.24			
**Cor Total**	23	272.96				
**R^2**		0.984				
**R^2_{pred}**		0.972				
**R^2_{aju}**		0.980				

Table S8-9 ANOVA RSM of mix M18

Source	gl	SC	CM	F	ϱ	Coef
**Model**	5	213.21	42.64	161.59	0.000	
**Linear**	2	186.13	60.75	230.21	0.000	
**Quadratic**	3	27.07	9.02	34.20	0.000	
**C1 * C3**	1	27.03	26.04	98.68	0.000	12.50
**C1 * C7**	1	0.04	0.04	0.16	0.696	0.50
**C3 * C7**	1	0.00	0.00	0.00	1.000	-0,00
**R. Error**	18	4.75	0.26			
**Cor Total**	23					
**R^2**		0.978				
**R^2_{pred}**		0.961				
**R^2_{aju}**		0.972				

Table S8-10 ANOVA RSM of mix M20

Source	gl	SC	CM	F	ϱ	Coef
**Model**	5	323.21	64.64	150.14	0.000	
**Linear**	2	248.03	72.33	168.00	0.000	
**Quadratic**	3	75.17	25.06	58.20	0.000	
**C1 * C3**	1	0.43	1.50	3.48	0.078	3.00
**C1 * C16**	1	20.74	10.67	24.77	0.000	-8.00
**C3 * C16**	1	54.00	54.00	125.42	0.000	18.00
**R. Error**	18	7.75	0.43			
**Cor Total**	23	330.96				
**R^2**		0.977				
**R^2_{pred}**		0.958				
**R^2_{aju}**		0.970				

Table S9. Analysis of variance of RSM, including diagnostic statistics for the affected fraction of *S. zeamais* for matrices with three compounds with contact toxic effect

Table S9-1 ANOVA RSM of mix MC1

Source	DF	SC	CM	F	ϱ	Coef
Model	5	152.33	30.47	49.85	0.000	
Linear	2	67.26	7.58	12.41	0.000	
Quadratic	3	85.07	28.36	46.40	0.000	
C1 * C24	1	78.45	67.43	110.35	0.000	17.14
C1 * C25	1	6.19	6.57	10.75	0.004	-6.29
C24 * C25	1	0.42	0.43	1.00	0.414	-1.60
R. Error	18	11.00	0.61			
Cor Total	23	163.33				
R^2		0.933				
R^2-pred		0.880				
R^2-adj		0.914				

Table S9-2 ANOVA RSM of mix MC7

Source	DF	SC	CM	F	ϱ	Coef
Model	5	164.37	32.87	52.60	0.000	
Linear	2	48.66	49.33	78.93	0.000	
Quadratic	3	115.72	38.57	61.72	0.000	
C26 * C36	1	101.46	81.87	130.99	0.000	22.42
C26 *C27	1	11.44	13.14	21.03	0.000	-3.92
C36 *C27	1	2.82	2.82	4.52	0.000	-1.97
R. Error	18	11.25	0.62			
Cor Total	23	175.62				
R^2		0.936				
R^2-pred		0.886				
R^2-adj		0.918				

Table S9-3 ANOVA RSM of mix MC6

Source	DF	SC	CM	F	ϱ	Coef
Model	5	257.83	51.57	36.40	0.000	
Linear	2	193.43	56.58	39.94	0.000	
Quadratic	3	64.40	21.47	15.15	0.000	
C25 * C36	1	24.01	35.04	24.74	0.000	14.50
C25 * C32	1	37.72	40.04	28.26	0.000	15.50
C36 *C32	1	2.67	2.67	1.88	0.187	4.00
R. Error	18	25.50	1.42			
Cor Total	23	283.33				
R^2		0.910				
R^2-pred		0.840				
R^2-adj		0.885				

Table S9-4 ANOVA RSM of mix MC8

Source	DF	SC	CM	F	ϱ	Coef
Model	5	51.50	10.30	21.81	0.000	
Linear	2	22.35	16.08	34.06	0.000	
Quadratic	3	29.16	9.72	20.59	0.000	
C28 * C27	1	0.73	0.014	0.03	0.865	-0.29
C28 * C11	1	28.05	26.04	55.15	0.000	12.50
C27 * C11	1	0.38	0.38	0.81	0.379	-0.52
R. Error	18	8.50	0.47			
Cor Total	23	60.00				
R^2		0.858				
R^2-pred		0.748				
R^2-adj		0.819				

Figure S7 Graphs of the fumigant toxicity interaction of the components of the mixtures using the median effect model of the law of mass action.

Figure S7-1a. M1- Median effect graph

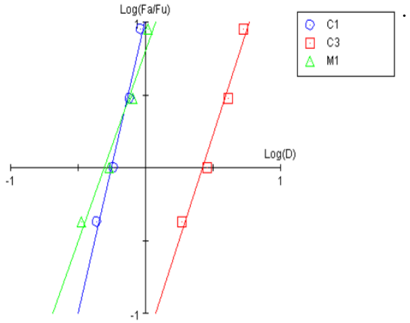

Figure S7-1b. M1- Combination Index Chart

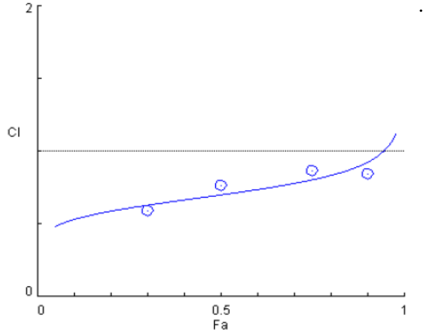

Figure S7-1c. M1- DRI log graph

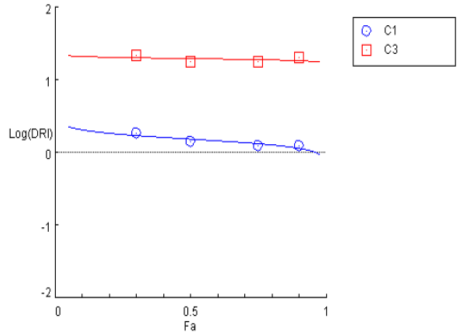

Figure S7-2a. M2- Median effect graph

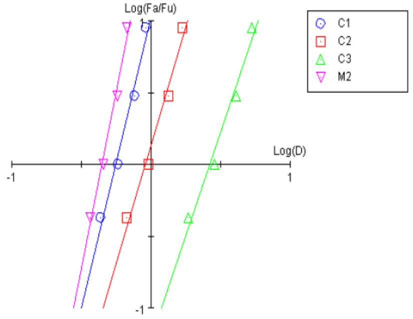

Figure S7-2b. M2- Combination Index Chart

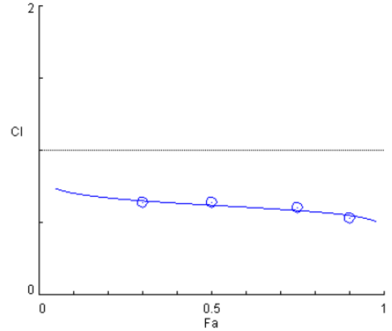

Figure S7-2c. M2- DRI log graph

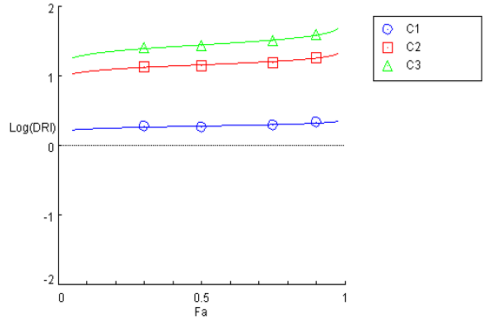

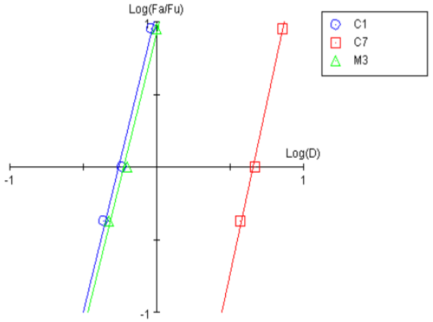

Figure S7-3a. M3- Median effect graph

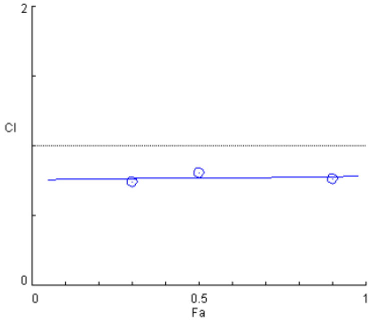

Figure S7-3b. M3- Combination Index Chart

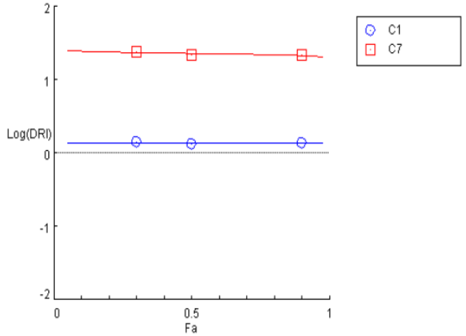

Figure S7-3c. M3- DRI log graph

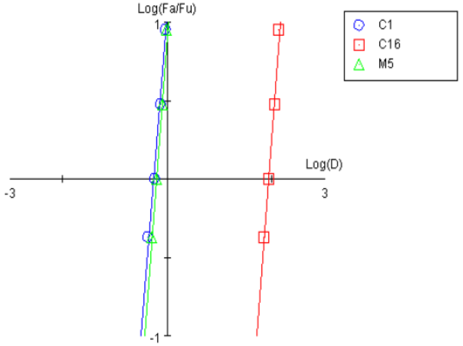

Figure S7-4a. M5- Median effect graph

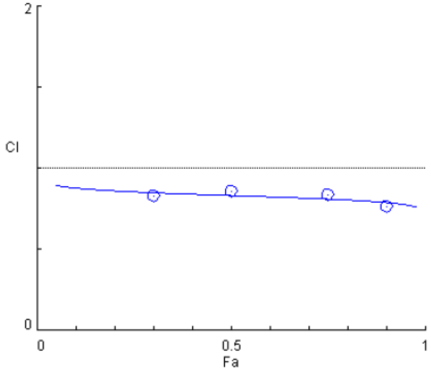

Figure S7-4b. M5- Combination Index Chart

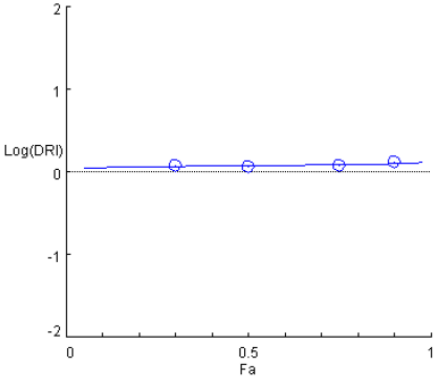

Figure S7-4c. M5- DRI log graph

Figure S7-5a. M12- Median effect graph

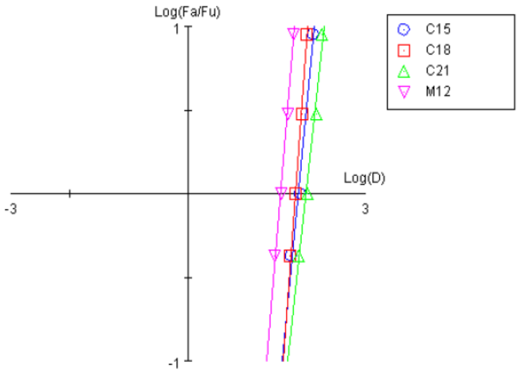

Figure S7-5b. M12- Combination Index Chart

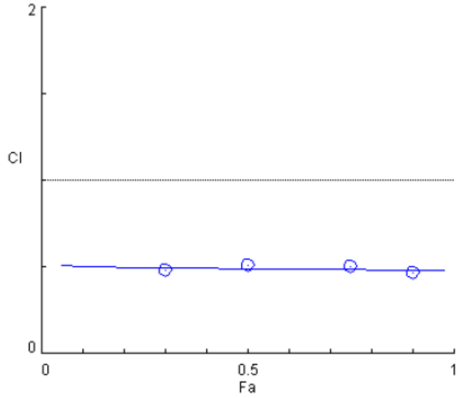

Figure S7-5c. M12- DRI log graph

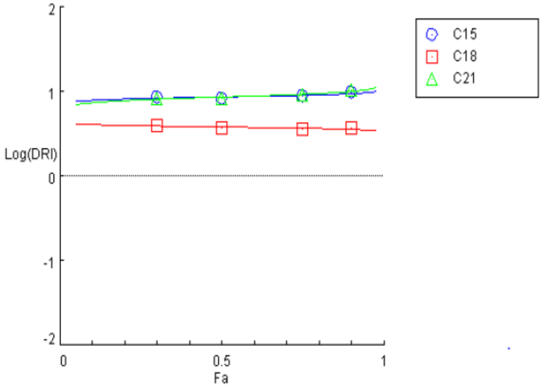

Figure S7-6a. M20- Median effect graph

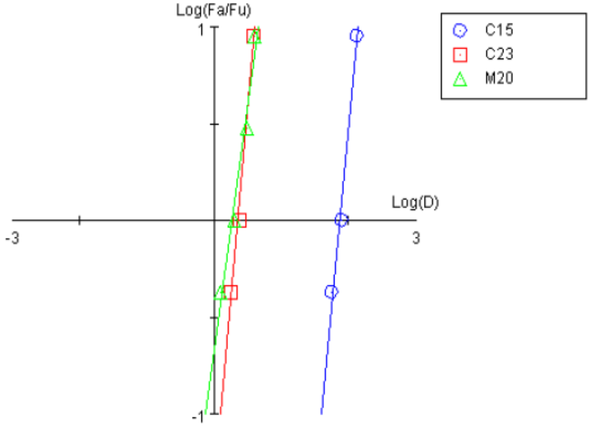

Figure S7-6b. M20- Combination Index Chart

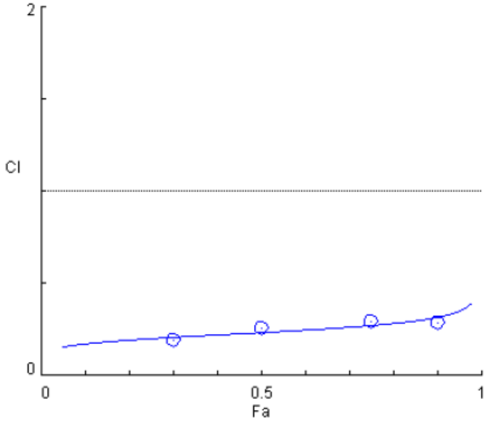

Figure S7-6c. M120- DRI log graph

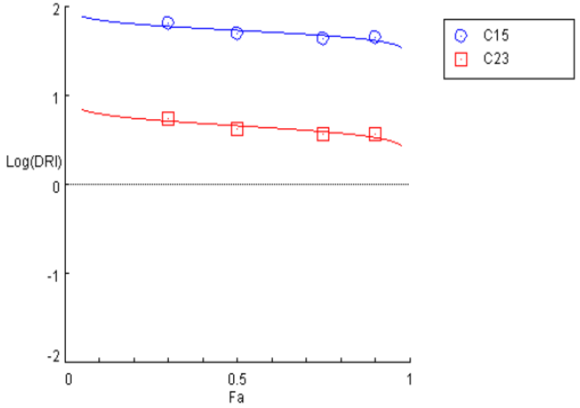

Figure S7-7a. M21- Median effect graph

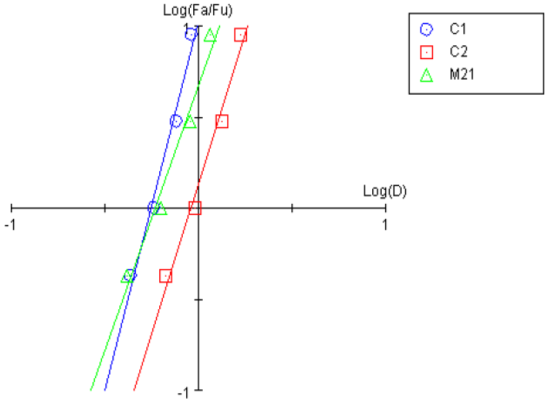

Figure S7-7b. M21- Combination Index Chart

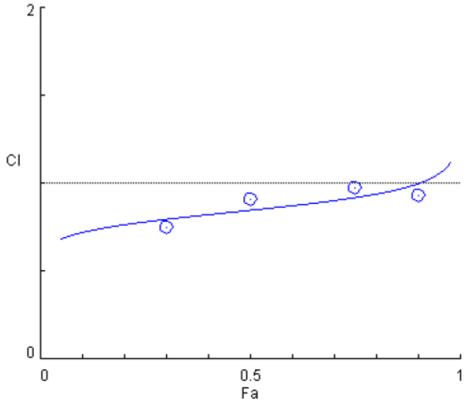

Figure S7-7c. M21- DRI log graph

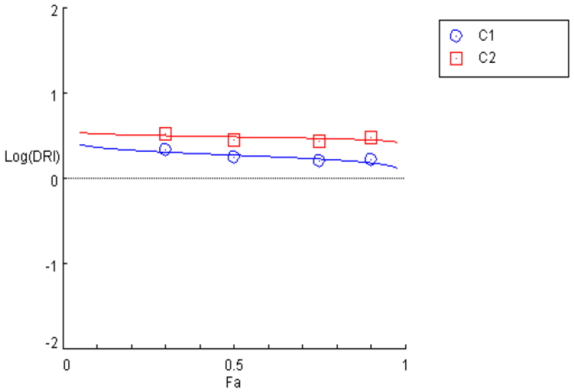

Figure S7-8a. MC1- Median effect graph

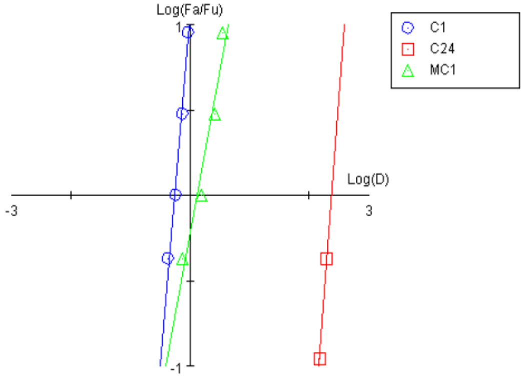

Figure S7-8b. MC1- Combination Index Chart

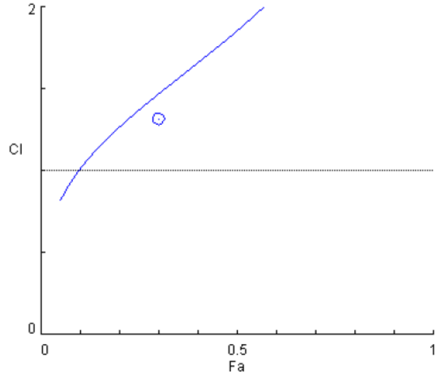

Figure S7-8c. MC1- DRI log graph

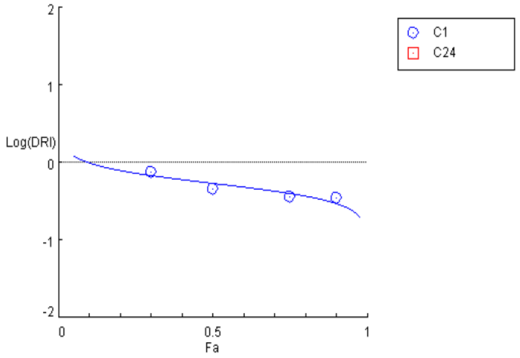

Figure S7-9a. MC8- Median effect graph

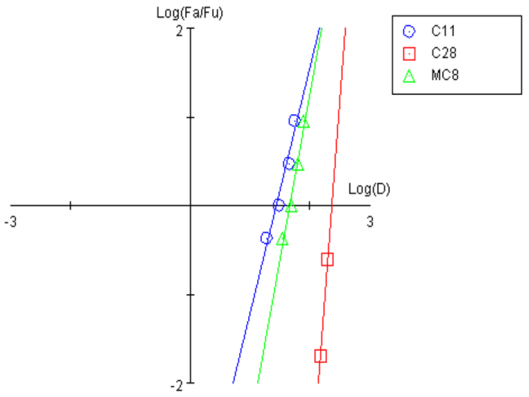

Figure S7-9b. MC8- Combination Index Chart

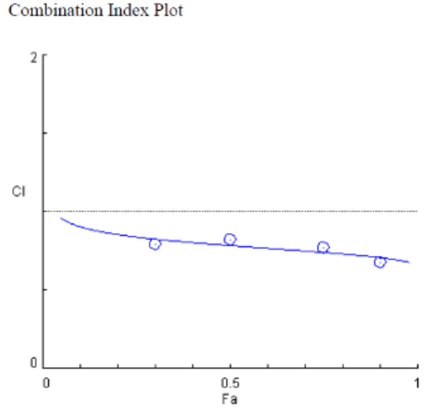

Figure S7-8c. MC8- DRI log graph

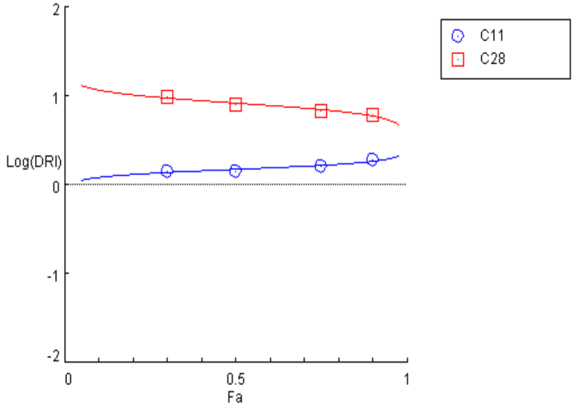

Figure S8 Graphs of the contact toxicity interaction of the components of the mixtures using the median effect model of the law of mass action.

Figure S8-1a. M1- Median effect graph

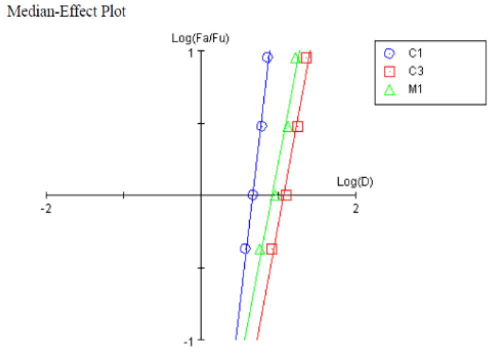

Figure S8-1b. M1- Combination Index Chart

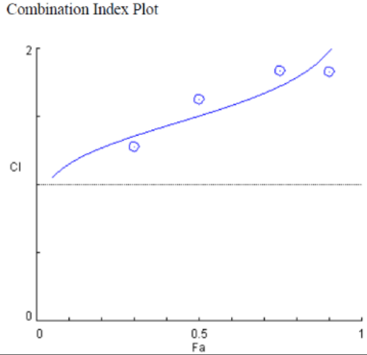

Figure S8-1c. M1- DRI log graph

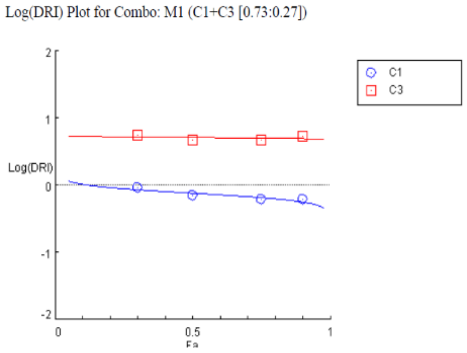

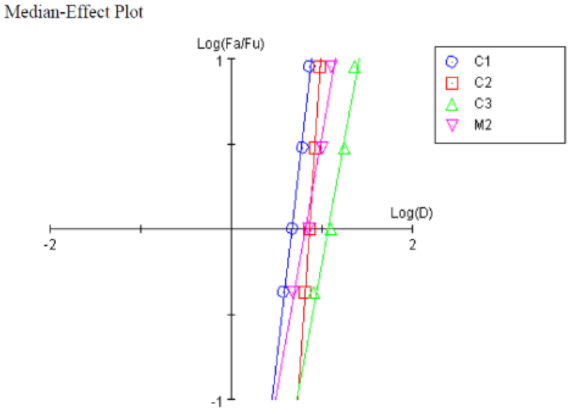

Figure S8-2a. M2- Median effect graph

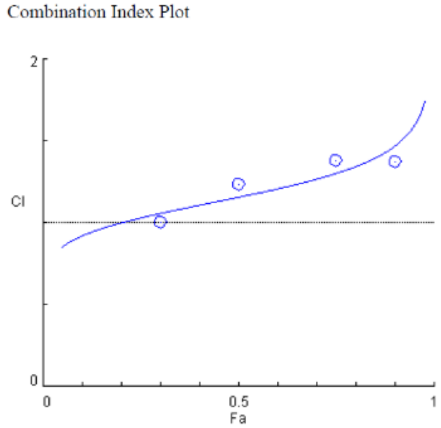

Figure S8-2b. M2- Combination Index Chart

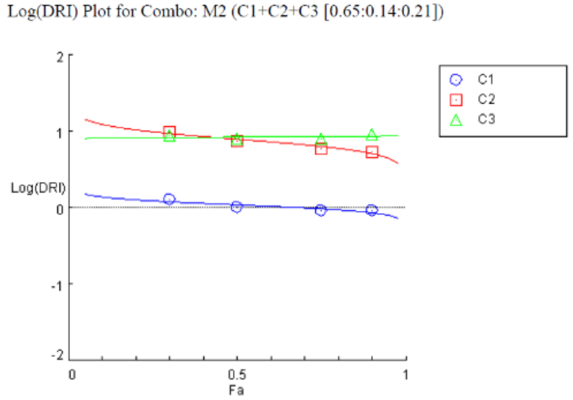

Figure S8-2c. M2- DRI log graph

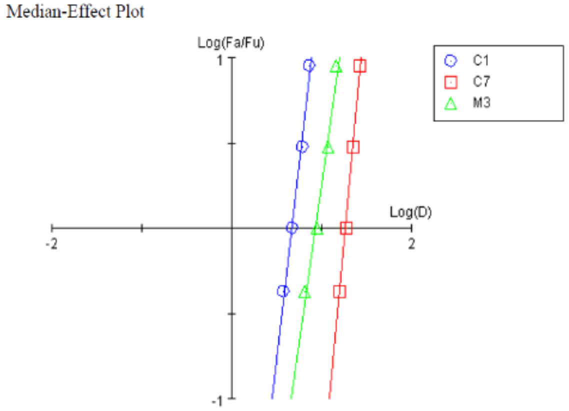

Figure S8-3a. M3- Median effect graph

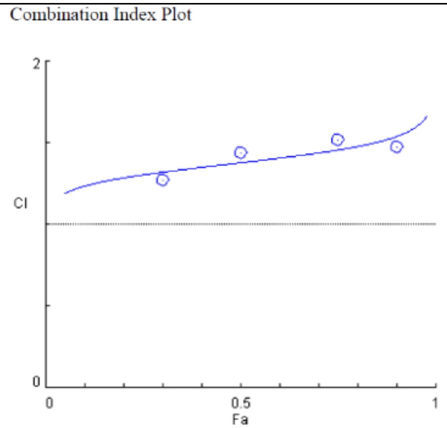

Figure S8-3b. M3- Combination Index Chart

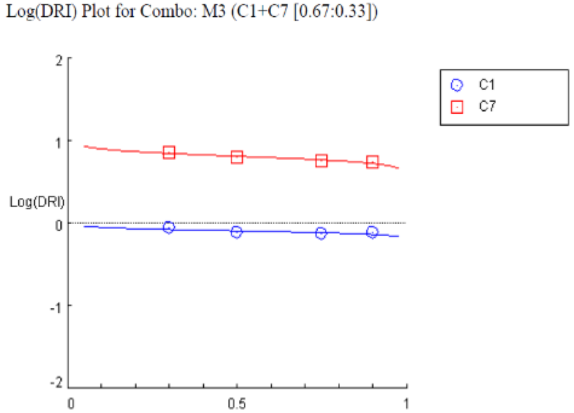

Figure S8-3c. M3- DRI log graph

Figure S8-4a. M5- Median effect graph

Figure S8-4b. M5- Combination Index Chart

Figure S8-4c. M5- DRI log graph

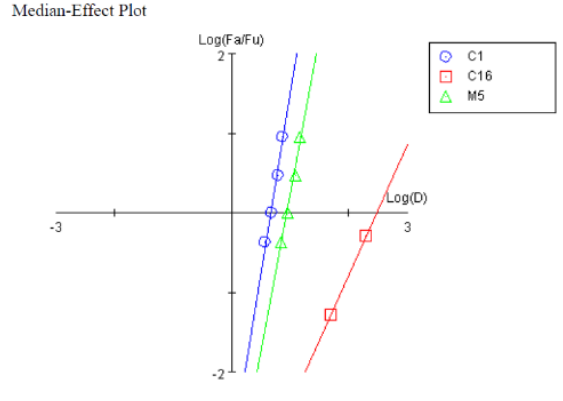

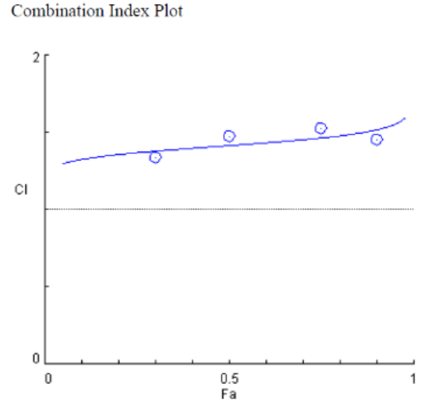

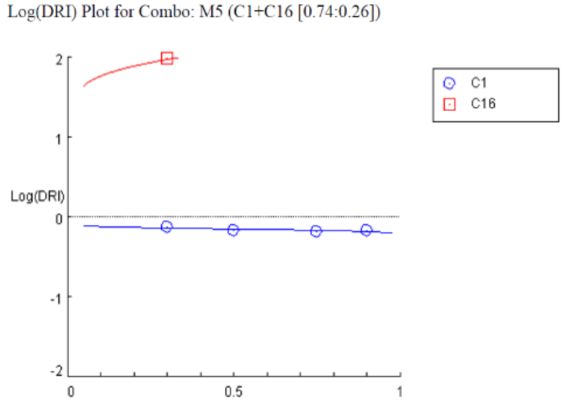

Figure S8-5a. M20- Median effect graph

Figure S8-5b. M20- Combination Index Chart

Figure S8-5c. M20- DRI log graph

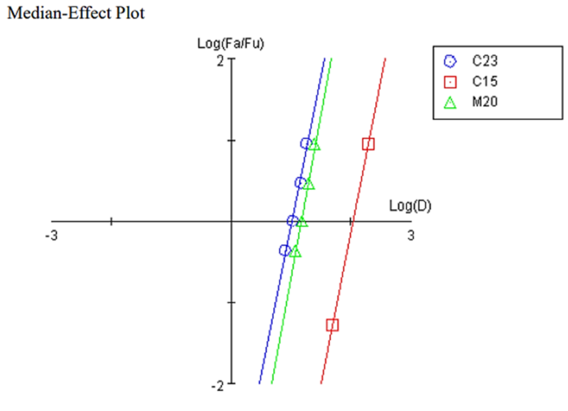

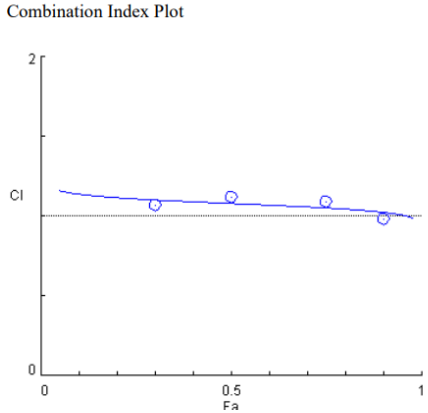

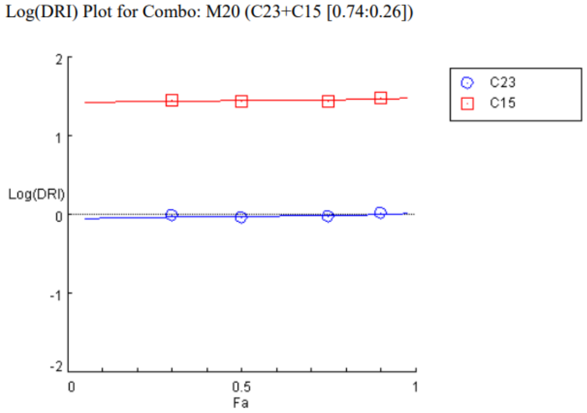

Figure S8-6a. M21- Median effect graph	*Figure S8-6b. M21- Combination Index Chart*	*Figure S8-6c. M21- DRI log graph*

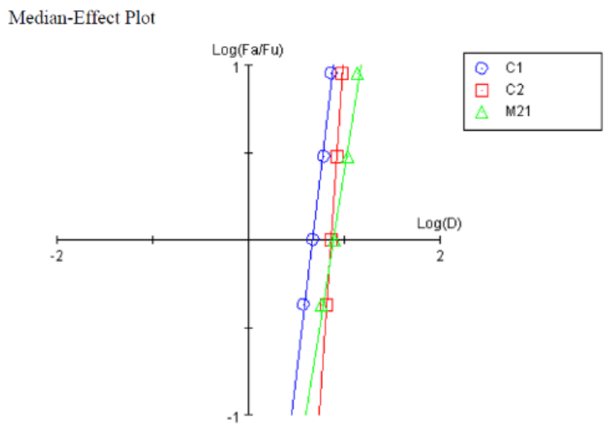

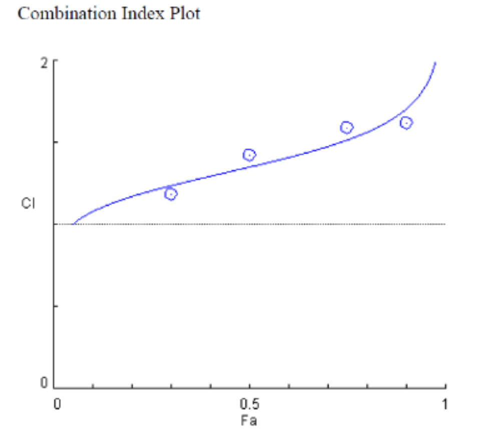

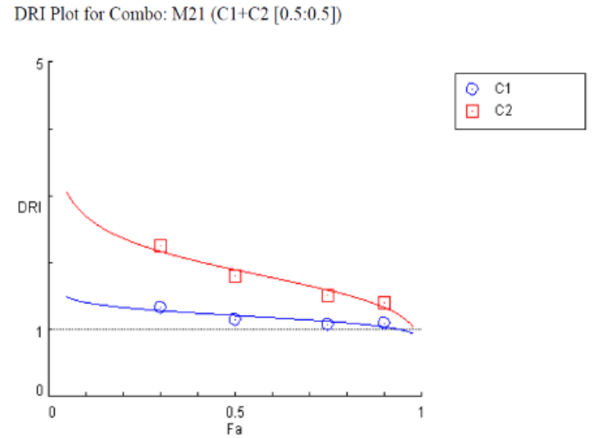

Figure S8-7a. MC1- Median effect graph	*Figure S8-7b. MC1- Combination Index Chart*	*Figure S8-7c. MC1- DRI log graph*

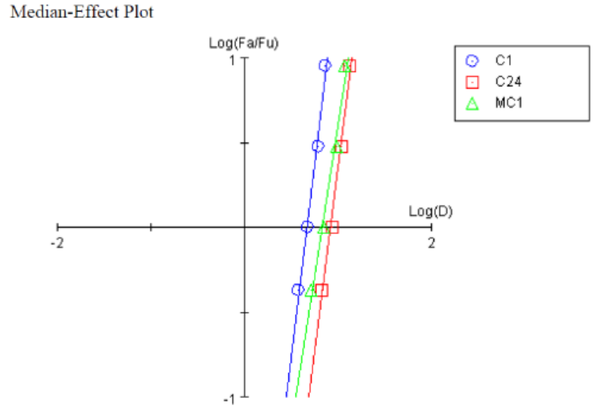

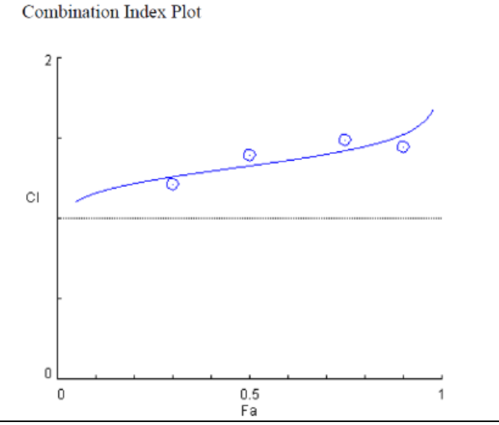

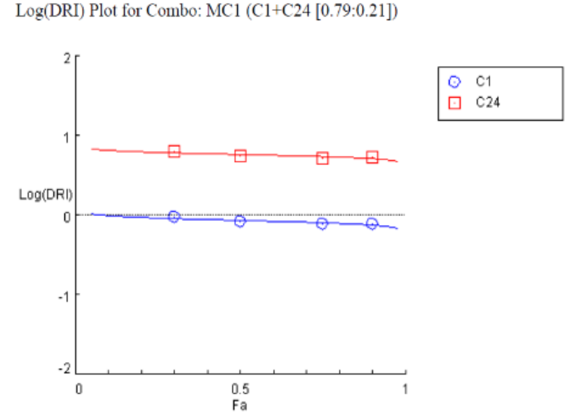

Figure S8-8a. MC6- Median effect graph

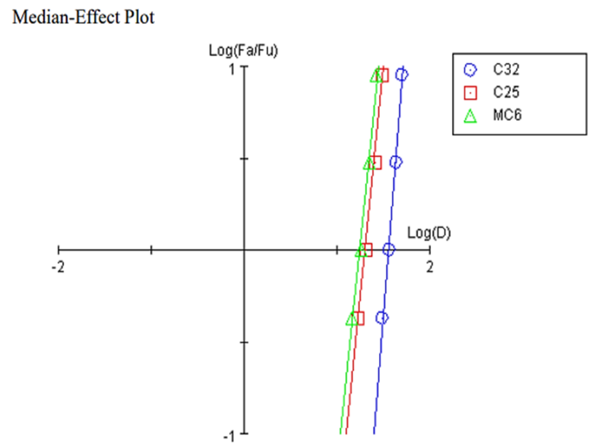

Figure S8-8b. MC6- Combination Index Chart

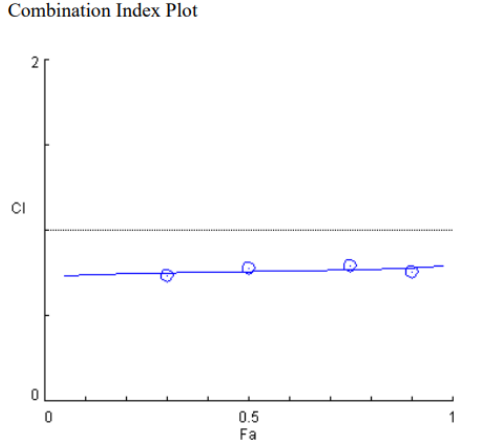

Figure S8-8c. MC6- DRI log graph

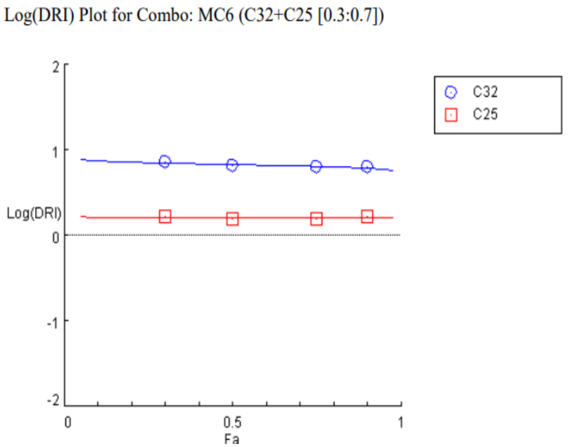

Figure S8-9a. MC8- Median effect graph

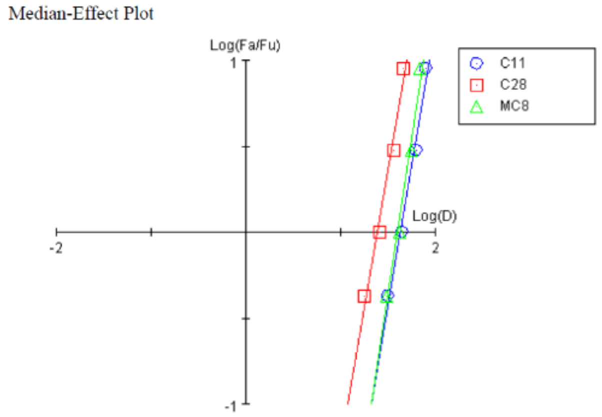

Figure S8-9b. MC8- Combination Index Chart

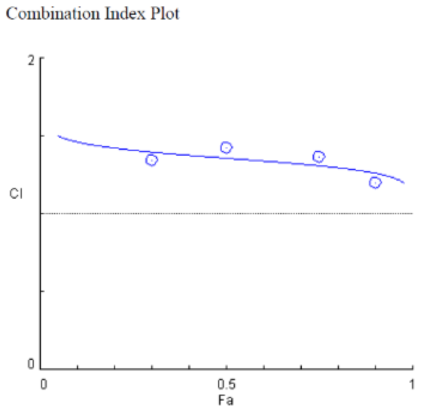

Figure S8-9c. MC8- DRI log graph

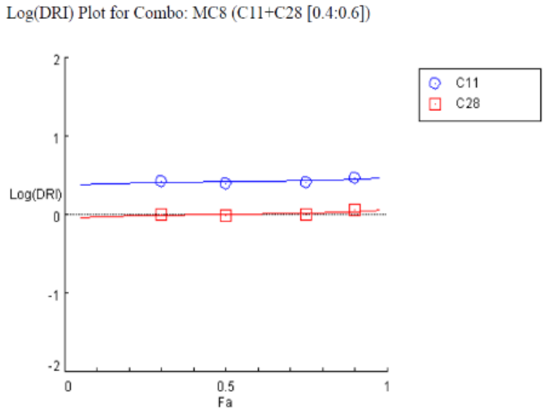

Table S10. characterization of Protein extract from *S. zeamais*

Protein extract	protein content g/mL	Characterized enzyme	Enzymatic activity U/mL	Specific enzymatic activity U/mg protein	Determined characteristics of the extract in assay			
					**Enzymatic activity U/mL**	**Time (min)**	***km***	**Substrate concentration *[S] ≈ 2 km* (mM)***
1	0.82 ± 0.04	AChE	2.50 ± 0.03	3.05 ± 0.04	0.31	35.0		0.60
2	0.84 ± 0.03	GST	29.18 ± 0.12	33.55 ± 0.03	0.59	8.0		1.60
3	0.84 ± 0.03	CAT	412.86 ± 0.05	491.50 ± 0.02	24.77	2.5		80.0

* Substrate concentration equivalent to 2 times the deaminated Michaelis-Menten constant

Figure S9. Dose-effect curves of *S. zeamais* AChE inhibition of the mixtures and their components

Figure S9.-1. Dose-effect curve VCs C2

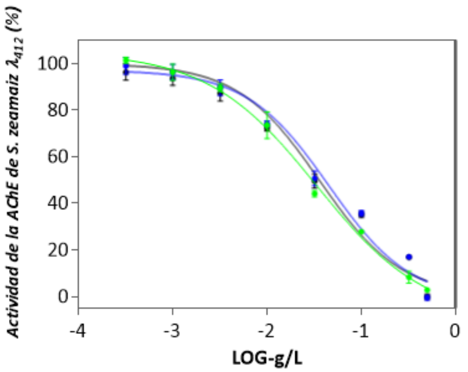

Figure S9.-2. Dose-effect curve VCs C3

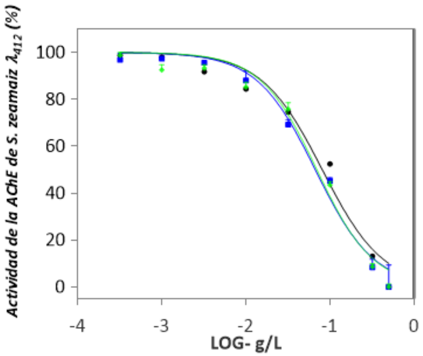

Figure S9-3. Dose-effect curve VCs C7

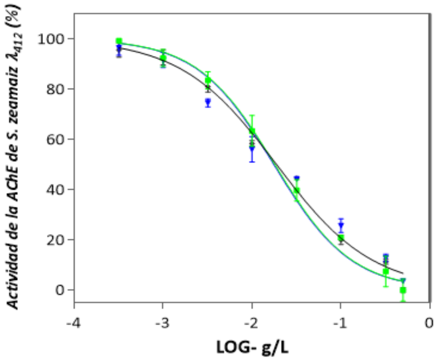

Figure S9-4. Dose-effect curve VCs C11

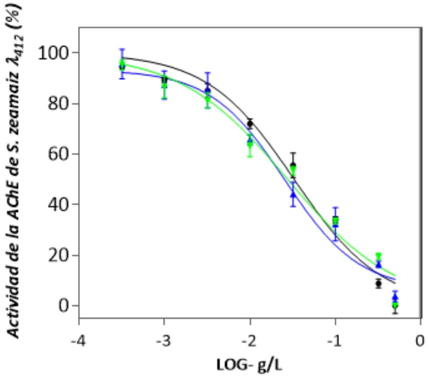

Figure S9-4. Dose-effect curve VCs C11

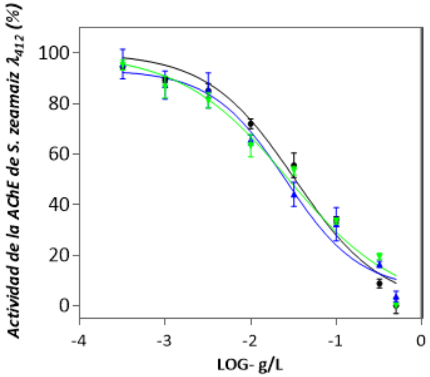

Figure S9-5. Dose-effect curve VCs C15

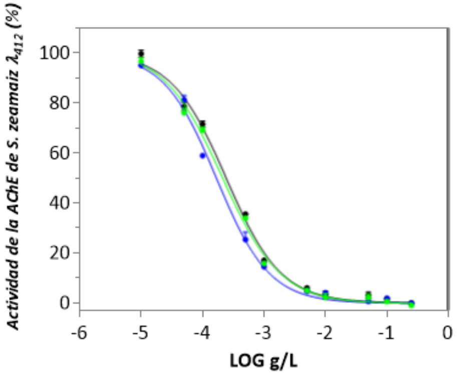

Figure S9-6. Dose-effect curve VCs C16

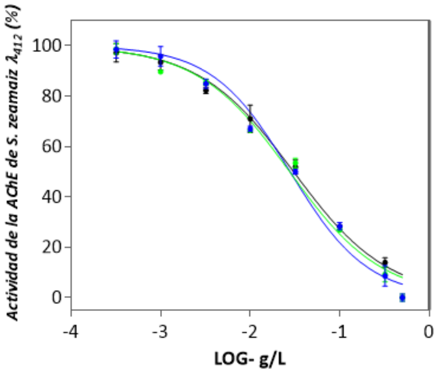

Figure S9-7. Dose-effect curve VCs C28

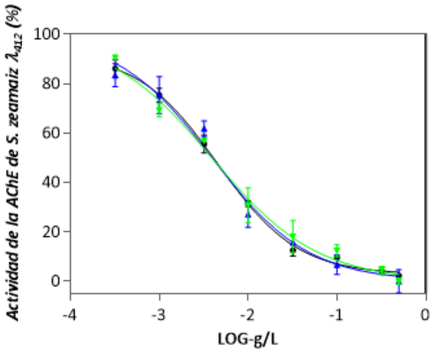

Figure S9-8. Dose-effect curve VCs C32

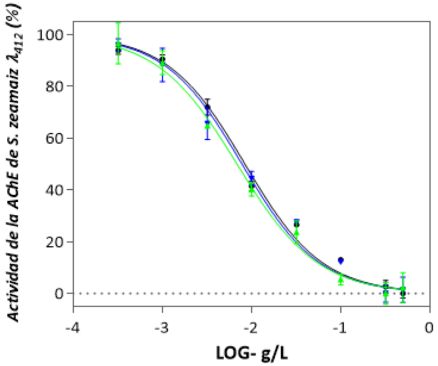

Figure S9-9. Dose-effect curve mixtures M5

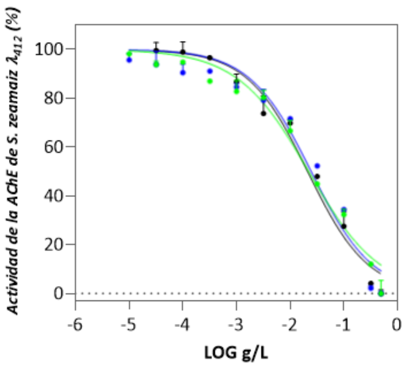

Figure S9-10. Dose-effect curve mixtures M12

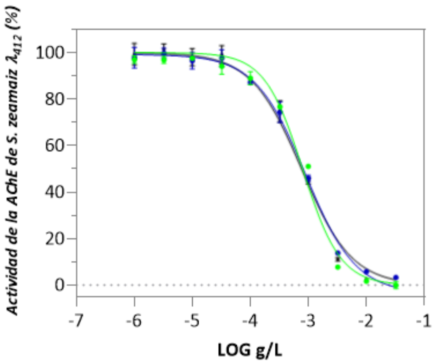

Figure S9-11. Dose-effect curve mixtures M20

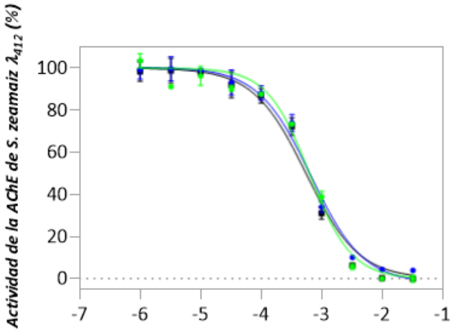

Figure S9-12. Dose-effect curve mixtures M21

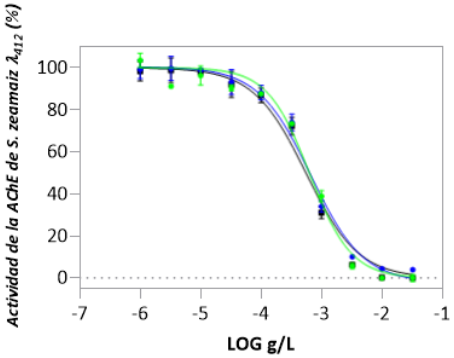

Figure S9-13. Dose-effect curve mixtures MC1

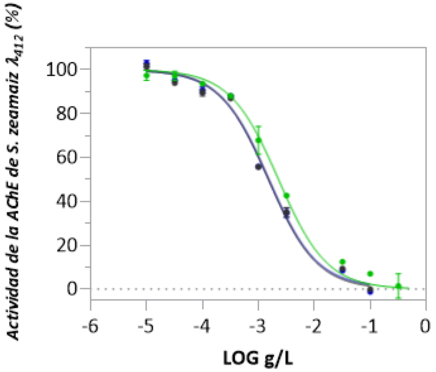

Figure S9-14. Dose-effect curve mixtures MC6

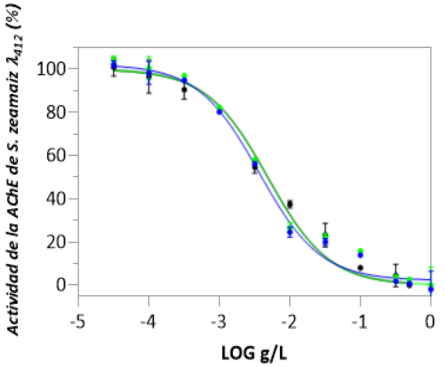

Figure S9-15. Dose-effect curve mixtures MC8

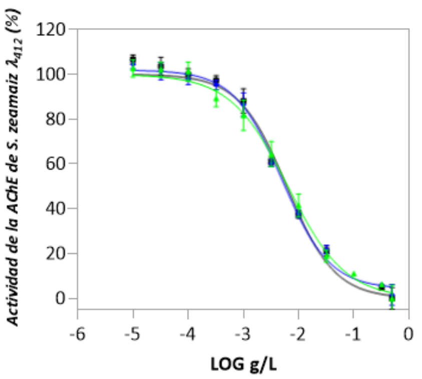

Figure S9-16. Dose-effect curve mixtures C+

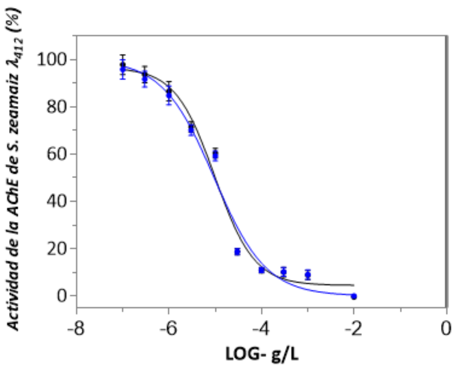

Figure S10. Lineweaver-Burk plots of the kinetic study of mixtures and their components on AChE on *S. zeamais*

Figure ***S**10-1. Lineweaver-Burk plots VCs C2*

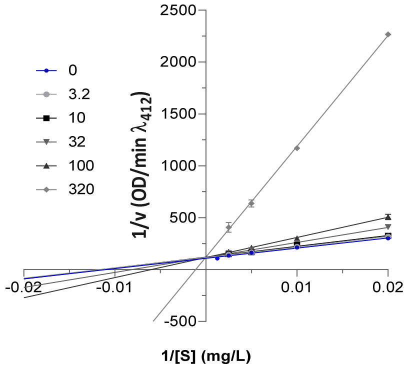

Figure ***S**10-2. Lineweaver-Burk plots VCs C3*

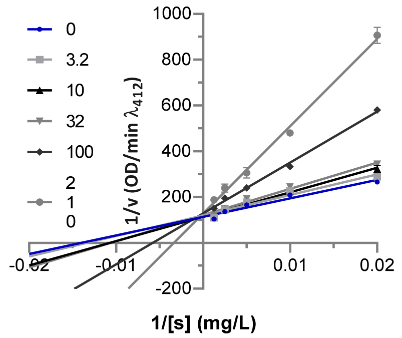

Figure ***S**10-3. Lineweaver-Burk plots VCs C7*

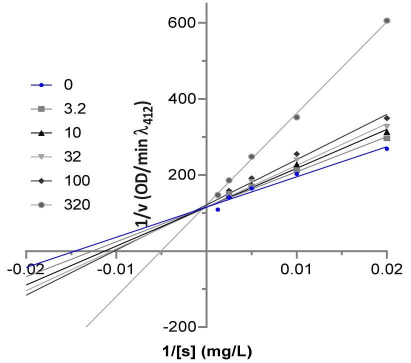

Figure ***S**10-4. Lineweaver-Burk plots VCs C11*

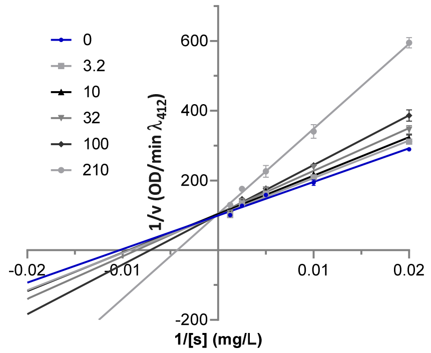

Figure ***S**10-5. Lineweaver-Burk plots VCs C15*

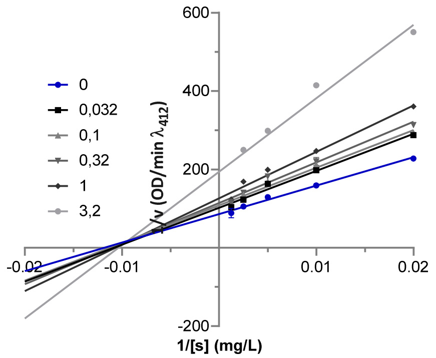

Figure ***S**10-6. Lineweaver-Burk plots VCs C16*

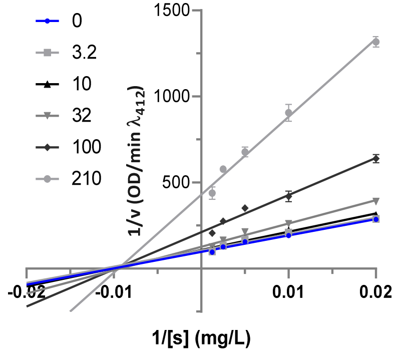

*Figure **S**10-7. Lineweaver-Burk plots VCs C29*

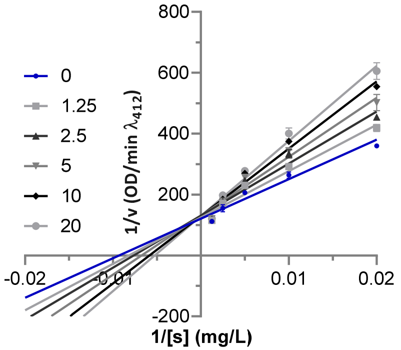

*Figure **S**10-8. Lineweaver-Burk plots VCs C33*

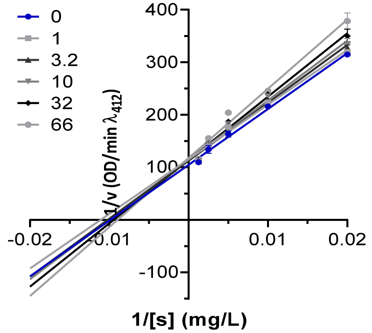

*Figure **S**10-9. Lineweaver-Burk plots Mixture M5*

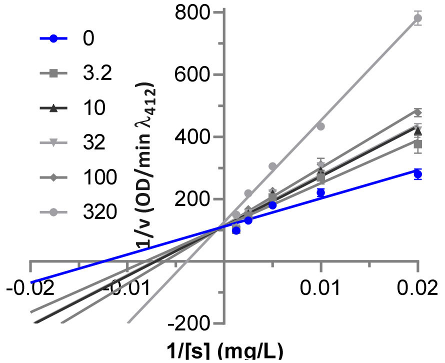

*Figure **S**10-10. Lineweaver-Burk plots Mixture M12*

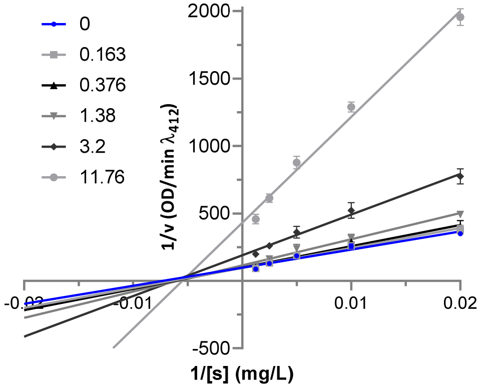

*Figure **S**10-11. Lineweaver-Burk plots Mixture M20*

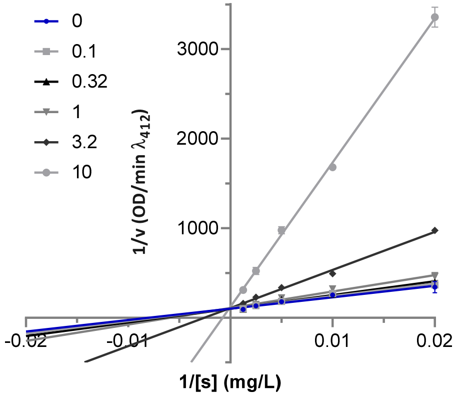

*Figure **S**10-12. Lineweaver-Burk plots Mixture M21*

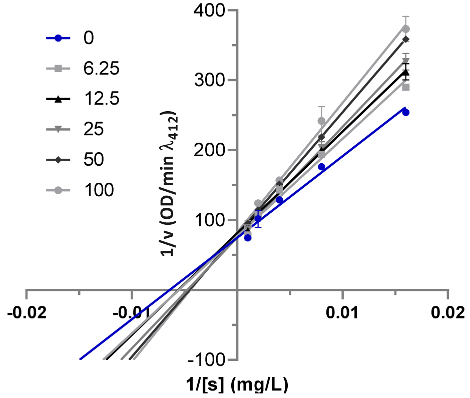

Figure S10-13. Lineweaver-Burk plots Mixture MC1

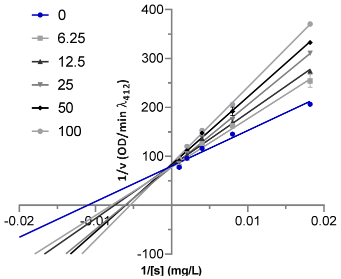

Figure S10-14. Lineweaver-Burk plots Mixture MC6

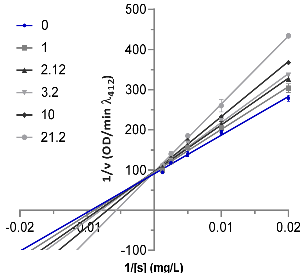

Figure S10-15. Lineweaver-Burk plots Mixture MC8

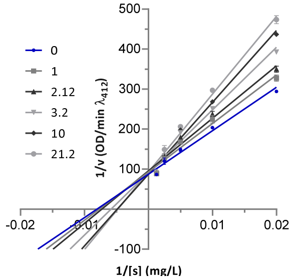

Figure S11 Graphs of the interaction of the components of the mixtures using the median effect model of the law of mass action.

Figure S11-1a. M5- Median effect graph

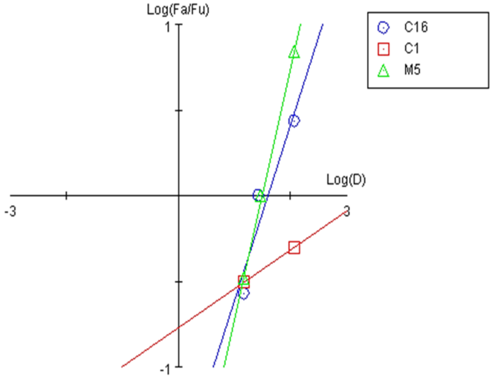

Figure S11-1b. M5- Combination Index Chart

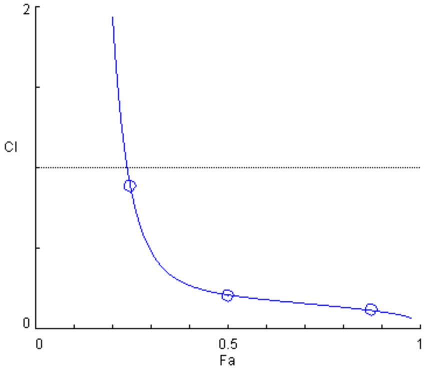

Figure S11-1c. M5- DRI log graph

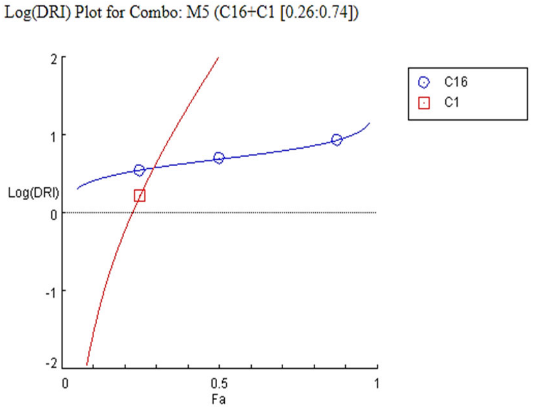

Figure S11-2a. M12- Median effect graph

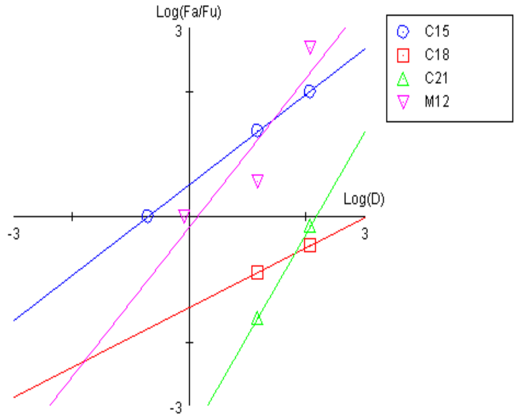

Figure S11-2b. M12- Combination Index Chart

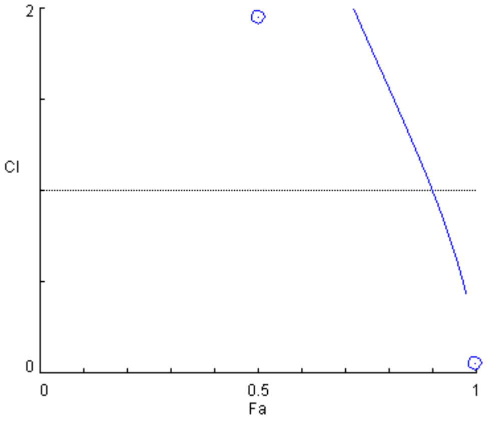

Figure S11-2c. M12- DRI log graph

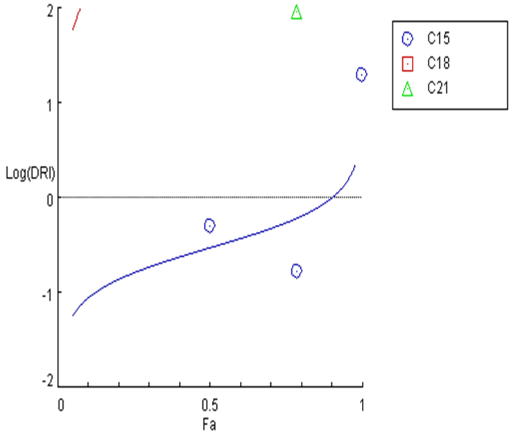

Figure S11-3a. M20- Median effect graph

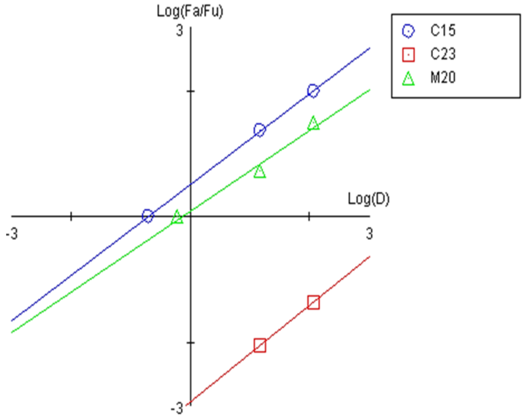

Figure S11-3b. M20- Combination Index Chart

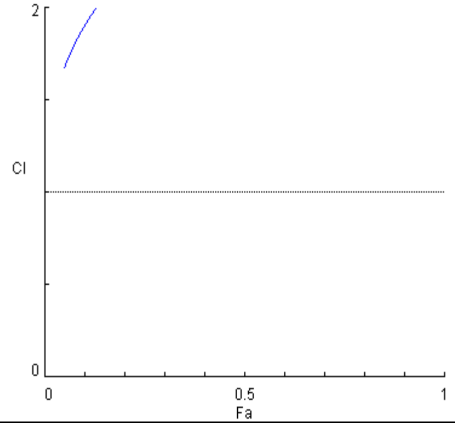

Figure S11-3c. M20- DRI log graph

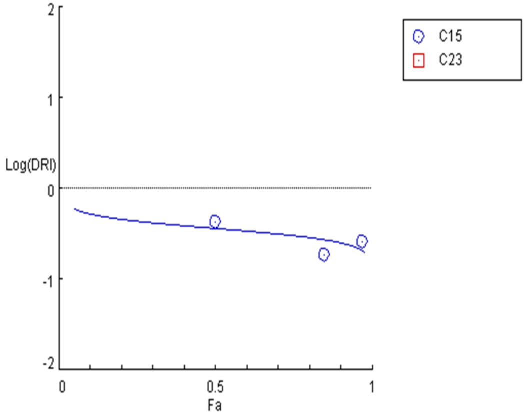

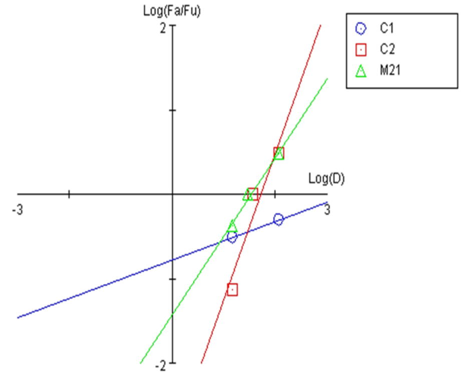

Figure S11-4a. M21- Median effect graph

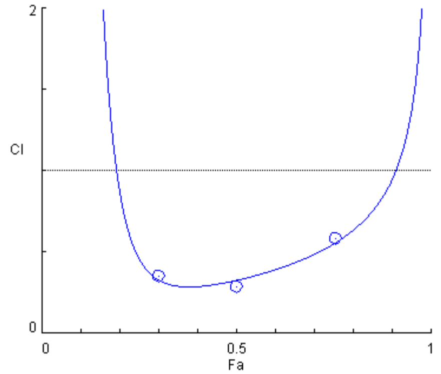

Figure S11-4b. M21- Combination Index Chart

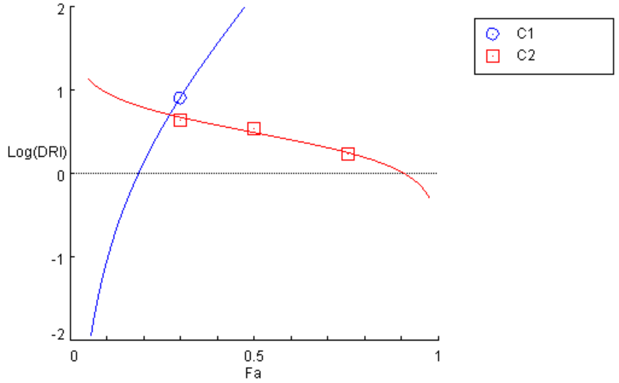

Figure S11-4c. M21- DRI log graph

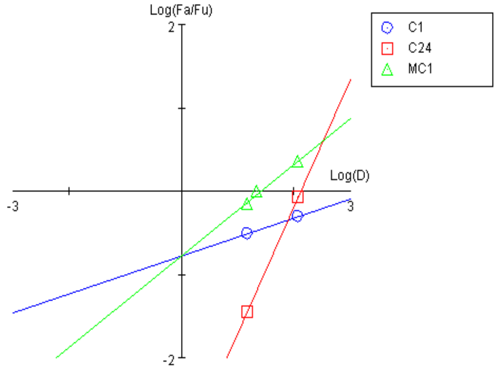

Figure S11-5a. MC1- Median effect graph

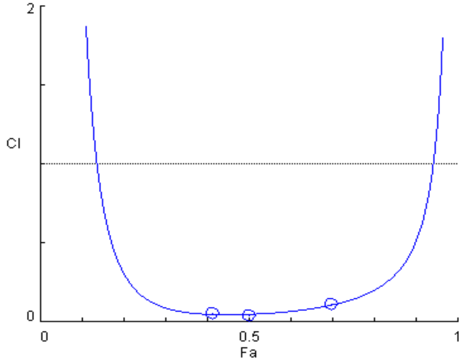

Figure S11-5b. MC1- Combination Index Chart

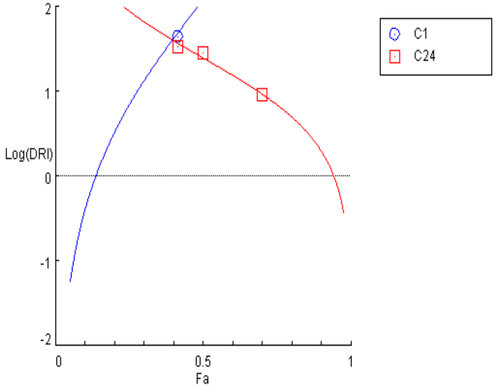

Figure S11-5c. MC1- DRI log graph

Figure S11-6a. MC6- Median effect graph

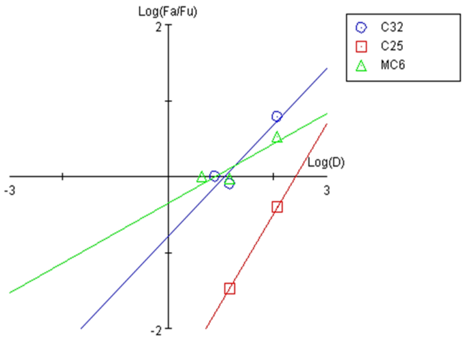

Figure S11-6b. MC6- Combination Index Chart

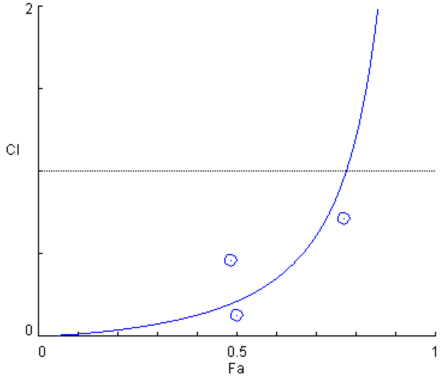

Figure S11-6c. MC6- DRI log graph

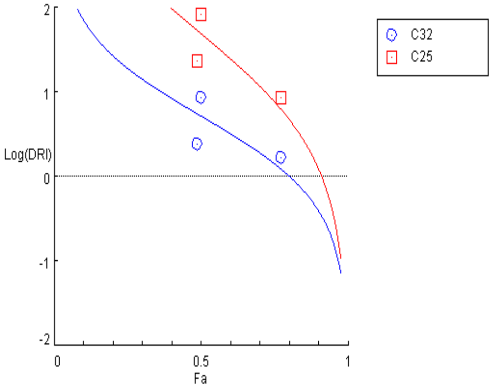

Figure S11-7a. MC8- Median effect graph

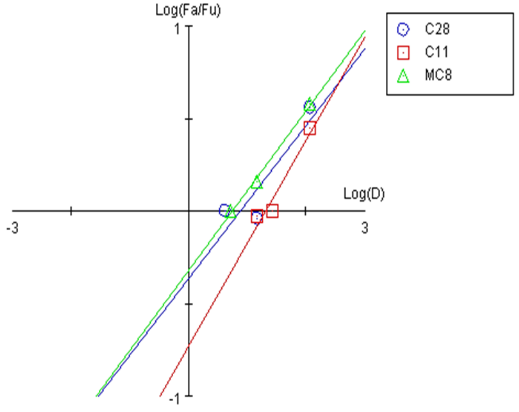

Figure S11-7b. MC8- Combination Index Chart

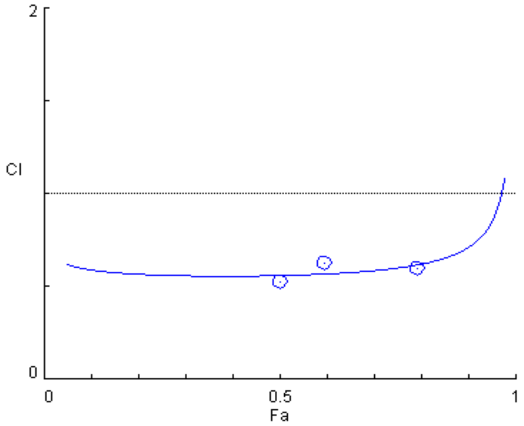

Figure S11-7c. MC8- DRI log graph

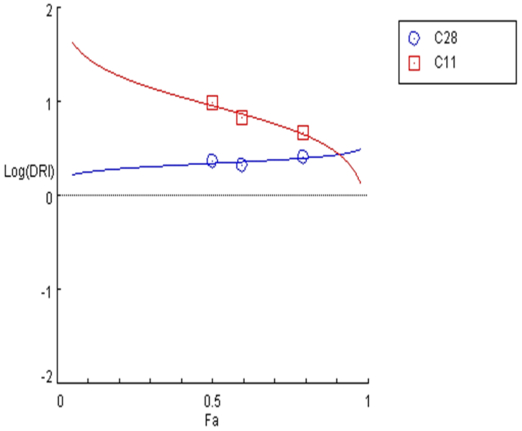

Disclaimer/Publisher's Note: The statements, opinions and data contained in all publications are solely those of the individual author(s) and contributor(s) and not of MDPI and/or the editor(s). MDPI and/or the editor(s) disclaim responsibility for any injury to people or property resulting from any ideas, methods, instructions or products referred to in the content.
